# Constitutive, endogenous, fluorescent membrane reporters for dynamic cell cycle analysis in *Bacillus subtilis*

**DOI:** 10.1128/aem.01126-26

**Published:** 2026-08-14

**Authors:** Jane H. Joncha, Sadie Ruesewald, Kehinde O. Adebiyi, Daniel B. Kearns, Stephen C. Jacobson

**Affiliations:** 1 Department of Chemistry, Indiana University Bloomington 1771, Bloomington, Indiana, USA; 2 Department of Biology, Indiana University Bloomington 1771, Bloomington, Indiana, USA; Michigan State University, East Lansing, Michigan, USA

**Keywords:** cytokinesis, PTS transport, growth, microfluidics, FtsZ, *Bacillus subtilis*, membrane label

## Abstract

Bacteria increase in biomass and divide, but determining precisely when cell division completes is technically challenging. To aid time-lapse imaging and cell-cycle tracking, we set out to identify a protein in *Bacillus subtilis* that, when fused with a fluorophore, would cause the membrane to fluoresce in a constitutive, uniform, and bright manner. A forward genetic transposon-based approach, combined with fluorescence-activated cell sorting, was used to identify a fluorescent fusion to the glucose PTS transmembrane transport protein PtsG with all desired properties. Moreover, PtsGΩmsfGFP was constitutive and neutral to growth under all conditions tested and also labeled membranes during sporulation. We used PtsGΩmsfGFP to track cell growth in microfluidic channels and modify the definition of cytokinesis as when membrane fluorescence reached a local maximum at the division plane, a definition further supported by fluorescence loss in photobleaching (FLIP) microscopy. Simultaneous imaging with a compatible fluorescent fusion to the cell division protein FtsZ indicated that FtsZ peak intensity occurred midway through septum constriction and that Z-ring recycling coincided with cytokinesis. We conclude that PtsGΩmsfGFP is a useful tool for membrane imaging and cell-cycle tracking. As such, we provide constructs with fluorophores that emit across the visible spectrum and various antibiotic resistance cassettes to facilitate deployment in *B. subtilis*.

**IMPORTANCE:**

Bacterial cells are fully divided when the new membrane separates the cytoplasm of each daughter. Reproducible staining of bacterial membranes with exogenous labels for fluorescence microscopy can be challenging, particularly during chemostatic growth in microfluidic devices. Here, we report that fusion of a fluorescent protein to the glucose transporter, PtsG, causes the membrane of *Bacillus subtilis* to emit bright, uniform fluorescence under a variety of conditions. We use PtsGΩmsfGFP to improve the definition of cytokinesis during microfluidic growth, and we note that a fluorescent PtsG fusion would likely facilitate fluorescent membrane staining in any organism, at least in theory.

## INTRODUCTION

Cellular growth occurs when cells coordinate an increase in biomass for division and is one of the most important, complex, and fundamental phenotypes of bacteria. Laboratory techniques for measuring bacterial growth each have their strengths and weaknesses. Spectrophotometry measures growth by light absorption of a culture and is facile but indirect, as it most directly measures biomass increase and not cell division *per se*. Viable counting involves dilution plating to track cell division events as a change in colony-forming units, but the results lag the actual measurement as time is needed for cells to grow to form visible colonies. Both techniques are often performed on batch cultures, where growth rates change over time either due to the depletion of nutrients or the accumulation of metabolic waste. Microscopic analysis of bacterial growth in microfluidic channels allows direct observation of cell division in a controlled chemostatic environment but requires specialized equipment (1–3). Moreover, determining when an individual cell has completed division can be challenging.

One type of cellular growth is binary fission, where a mother cell divides in the middle to produce two identical daughter cells. The process begins once the mother reaches a particular mass, and treadmilling protofilaments of the cell division protein FtsZ condense into a ring at or near the geometric midpoint of the cell (4–8). FtsZ continues to be recruited to the midcell until a threshold is reached, at which point a membrane-associated complex of peptidoglycan synthesis machinery, called the divisome, is recruited and activated (9–11). Next, the membrane constricts, and the FtsZ ring contracts (6, 12, 13). Once the constricting membrane fuses into a septum, cytokinesis, or the separation of daughter cell cytoplasms, is complete. In many gram-positive bacteria, like *Bacillus subtilis*, daughter cell separation begins after the completion of the division septum such that one often cannot tell if or when cell division has occurred by phase contrast microscopy.

In prior microfluidic experiments with *B. subtilis*, our group used a definition of cytokinesis based on a 20% reduction in the fluorescence intensity of a cytoplasmic protein (14). We recognized that defining cytokinesis by cytoplasmic fluorescence loss, however, might be complicated by the small scale of bacteria, the resolution limit of fluorescence microscopy, and the fact that cell separation does not necessarily have to occur after cell division in *B. subtilis*. Thus, we considered that cytokinesis could be more directly and accurately measured as an increase in membrane fluorescence during septum formation. As commonly used membrane stains such as FM4-64 and TMA-DPH are difficult to deliver evenly over long periods of time, we sought to generate a constitutively expressed membrane-anchored fluorescent protein. Preliminary reverse genetic approaches to make either natively expressed or inducible alleles of artificial fluorescent fusions to the transmembrane segment of either SwrB (15) or EzrA (16) failed to localize to the membrane for unknown reasons (data not shown).

Here, we use a forward genetic approach to identify a fusion of a fluorescent protein to the PTS transport component PtsG, which is brightly fluorescent, constitutive, and localizes to the cell membrane. Moreover, the PtsG fusion is not deleterious to growth in either a variety of batch media conditions or chemostatic microfluidic channels. Microfluidic monitoring of PtsGΩmsfGFP fluorescence led to a modified definition of cytokinesis in which the local fluorescence intensity of the invaginating membrane reached a maximum. When compared to the previous definition, we found that the previous 20% loss of cytoplasmic fluorescence preceded cytokinesis by a quarter of the cell cycle and, while precise, should be revised to a 40% decrease to more accurately coincide with septum formation. Membrane labeling also refined the analysis of FtsZ dynamics, indicating that mCherry-FtsZ continues to intensify after membrane invagination is initiated to a maximum, after which FtsZ disassembles to extinction at or near the point of membrane transection. We conclude that PtsGΩmsfGFP is a useful and versatile tool for membrane staining that improves the resolution of microscopic growth analyses.

## RESULTS

### A forward genetic approach to isolate msfGFP fusions with membrane localization

**Fig 1:**
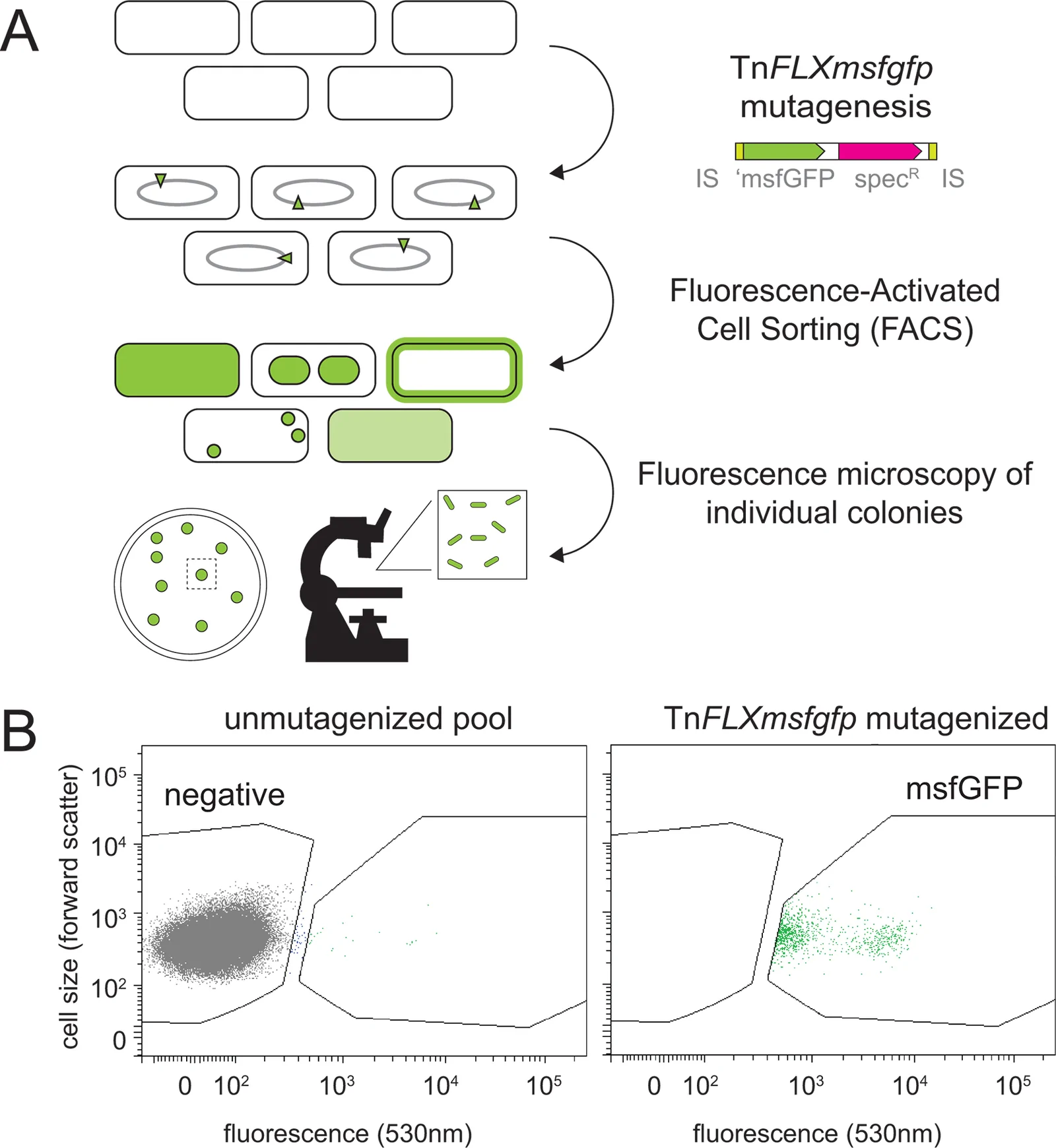
FACS-based strategy for isolating random translational fusions to msfGFP. (**A**) A cartoon outline of the overall screening strategy. Wild-type cells were mutagenized with a transposon carrying the spectinomycin antibiotic resistance cassette and an inward-facing msfGFP gene lacking a ribosome-binding site and translational start. IS elements are indicated in yellow, the msfGFP gene as a green arrow, and the spectinomycin resistance gene as a magenta arrow. Thus, cells could only become fluorescent if the gene encoding msfGFP was inserted in frame into a gene that was being actively transcribed. To isolate cells containing fluorescent fusions, the pool was analyzed by fluorescence-activated cell sorting, and cells were selectively isolated if their fluorescence exceeded a threshold gate (Fig. S1). Individual colonies were isolated from the pool of cells with green fluorescence, manually grown, and observed by fluorescence microscopy. Finally, the location of each transposon was determined by inverse PCR and sequencing. (**B**) FACS sorting data of the transposon mutagenized pool. Left: A FACS sorting of unmutagenized wild type to establish the boundaries of non-fluorescent cells. Each gray dot represents a non-fluorescent individual. Right: A FACS sorting of Tn*FLXmsfgfp* mutagenized wild type. Each green dot indicates an individual with fluorescence in excess of a threshold gate. Note that there were also many non-fluorescent individuals in this population (gray circles) that were omitted due to an excess of data points. Ultimately, ~2,000 green individuals were sorted from ~40,000,000 cells in the total population so that candidates occurred at a frequency of 0.005%.

To identify a candidate fusion that provides intense, constitutive, and evenly distributed membrane fluorescence, cells were mutagenized with a transposon capable of generating 3′ translational fusions to monomeric super-folder green fluorescent protein (msfGFP) (Fig. 1A). Briefly, a plasmid carrying the spectinomycin-selectable Tn*FLXmsfgfp* transposon was transformed into wild-type DK1042 cells and grown at room temperature for extra-chromosomal maintenance of the delivery vehicle. Colonies were selected and grown to high density in lysogeny broth (LB) at 37°C, a temperature that is non-permissible for plasmid maintenance, and then plated for high-density single colonies on plates containing spectinomycin at the same temperature. The colonies were pooled, back-diluted, and grown to mid-exponential phase. Fluorescent individuals were separated by fluorescence-activated cell sorting (FACS) (Fig. S1). Specifically, all cells with fluorescence intensity more than a threshold established by comparison to a non-mutagenized, non-fluorescent control were pooled (Fig. 1B) and dilution-plated for single colony-forming units. One hundred colonies were individually screened by fluorescence microscopy, and 70 were discarded, as they failed to give detectable signals. The location of the transposon insertion was determined in the 30 remaining isolates, and after eliminating the siblings, 12 strains were reserved for further analysis (Table 1).

**TABLE 1:** Genes in which *TnFLXmsfgfp* had inserted and produced fluorescence

Gene	Annotation	Pattern	Insertion tag^*a*^
*ccpA*	Carbon catabolite regulator	Nucleoid	TAGAGCTTA
*glyS*	Glycyl-tRNA synthetase β-subunit	Cytoplasmic	TATATAGAA
*guaB*	Inosine monophosphate dehydrogenase	Cytoplasmic	TACAGATTA
*manP*	PTS enzyme IIABC (mannose)	Cytoplasmic	TACCGGTCT
*motA*	Flagellar stator protein	Cytoplasmic	TATTTAAGG
*msmX*	ATP-binding subunit, ABC transport	Punctate	TACATGATG
*ptsG*	PTS enzyme IIABC (glucose)	Membrane	TAATGAATG
*ptsG*	PTS enzyme IIABC (glucose)	Membrane	TATTTACAA
*yidC1*	Protein translocase	Cytoplasmic	TACCAACTA
*yknW*	Unknown function	Cytoplasmic	TATTATTAG
*ywdK*	Unknown function	Membrane	TAAGTGTGA
*yybN*	Unknown function	Punctate	TAAAGCAGC

^a^ insertion tag indicates the TA followed by seven bases at which the transposon had inserted.

**Fig 2:**
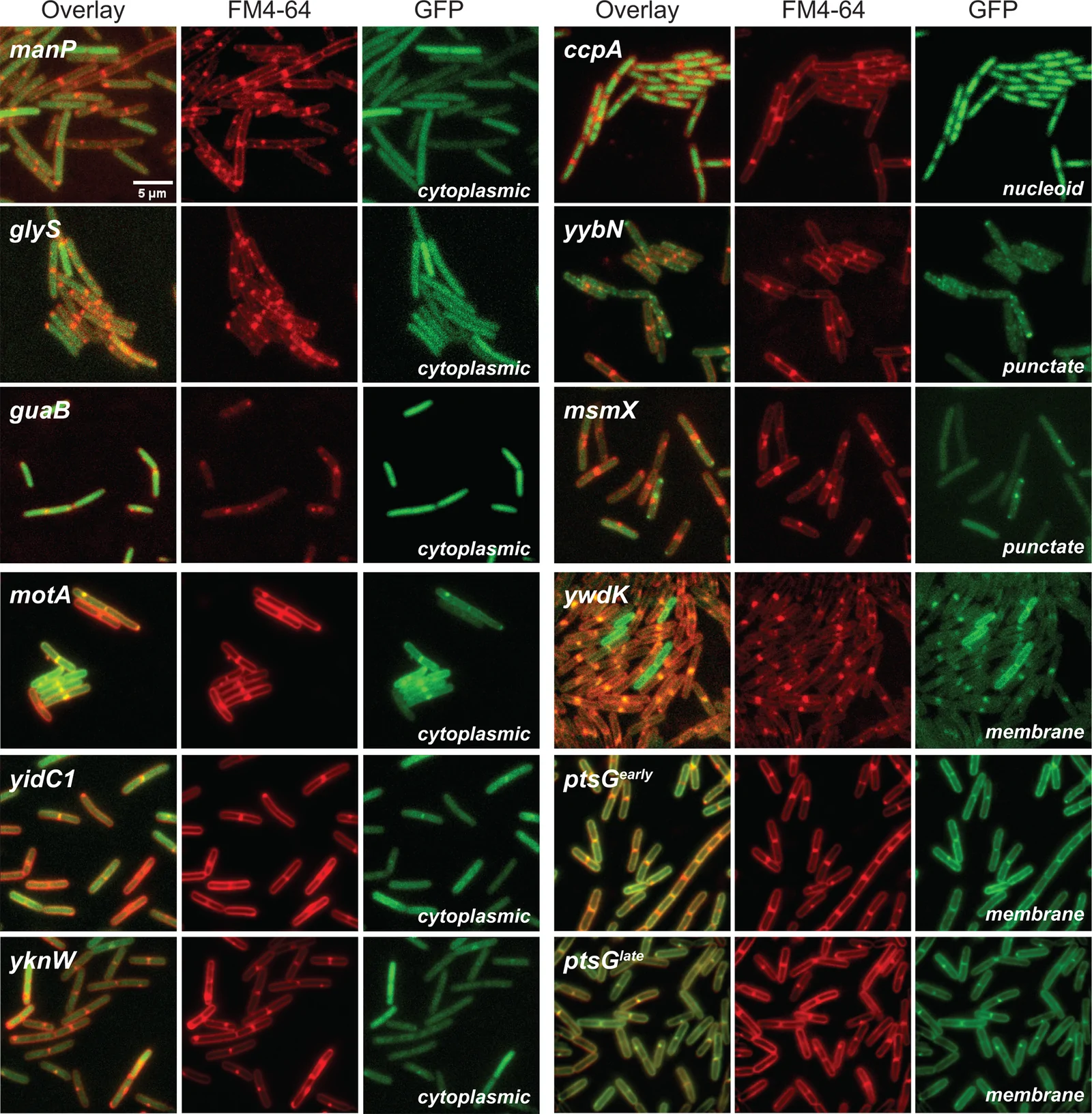
Cells expressing msfGFP fusions displayed a variety of subcellular localization patterns. Fluorescence micrographs of cells expressing an msfGFP fusion to the gene indicated in the upper left of each panel series. The left panel is an overlay of the membrane stain FM4-64 (false-colored red, exposure time 500 ms) and the fluorescence from the msfGFP fusion protein (false-colored green, exposure time 500–700 ms). Each localization pattern was characterized as cytoplasmic, aggregate, nucleoid, punctate, and membrane accordingly in the bottom right of each panel series (Table 1). The following strains were used for the generation of this figure: *manP* (DB1847), *glyS* (DB2138), *guaB* (DB2142), *motA* (DB2141), *yidC1* (DB2372), *yknW* (DB2136), *ccpA* (DB1701), *yybN* (DB1948), *msmX* (DB2140), *ywdK* (DB2137), *ptsG^early^* (DB1567), and *ptsG^late^* (DB1401). Scale bar is 5 μm.

Each of the unique insertions produced a variety of localization patterns in their respective strains. Six of the strains exhibited bright fluorescence that was diffuse in the cytoplasm (Fig. 2, cytoplasmic). Two generated fusions to the predicted soluble proteins, GlyS (glycyl-tRNA synthetase) and GuaB (inosine monophosphate dehydrogenase). A third was fused to the multi-pass transmembrane protein ManP (mannose phosphotransferase system) but was fused early in the protein prior to the first transmembrane segment. The final three strains had fusions to proteins predicted to be in the membrane: MotA (flagellar motor protein A), YidC1 (Sec-independent protein translocase), and YknW (unknown function). We infer that the cytoplasmic localization pattern likely indicates dysfunctional fusions, as functional fusions to MotA appear as disparate puncta on the membrane (17), and the msfGFP fusion after a number of transmembrane segments in YidC1 might have been expected to have membrane localization (18, 19).

One fusion in CcpA, the DNA-binding carbon catabolite regulator protein (20), had a localization pattern reminiscent of the nucleoid (Fig. 2, nucleoid), and the fluorescence co-localized with DAPI staining (Fig. S2). Two other fusions in YybN (unknown function) and MsmX (an ATP-dependent transport protein) (21, 22) exhibited punctate localization patterns (Fig. 2, punctate). Finally, three fusions appeared to give membrane localization (Fig. 2, membrane). Fusion of msfGFP to YwdK (unknown function) strongly labeled the membrane but did so only in a minority subpopulation. The remaining two membrane-localized fusions were inserted at two locations in PtsG (enzyme IICBA of the glucose phosphotransferase system), one early (“PtsG^early^” inserted after Val100) in the coding sequence and one late (“PtsG^late^” after Val669). We conclude that the random Tn*FLXmsfgfp* mutagenesis approach for generating fluorescent protein fusions was successful and that the PtsGΩmsfGFP fusions were good candidates for a constitutive, endogenous membrane stain. We selected PtsG^early^ΩmsfGFP (hereafter referred to as PtsGΩmsfGFP) for subsequent study as its fluorescence was brighter than PtsG^late^ΩmsfGFP.

### PtsGΩmsfGFP does not appear to confer a growth defect

**Fig 3:**
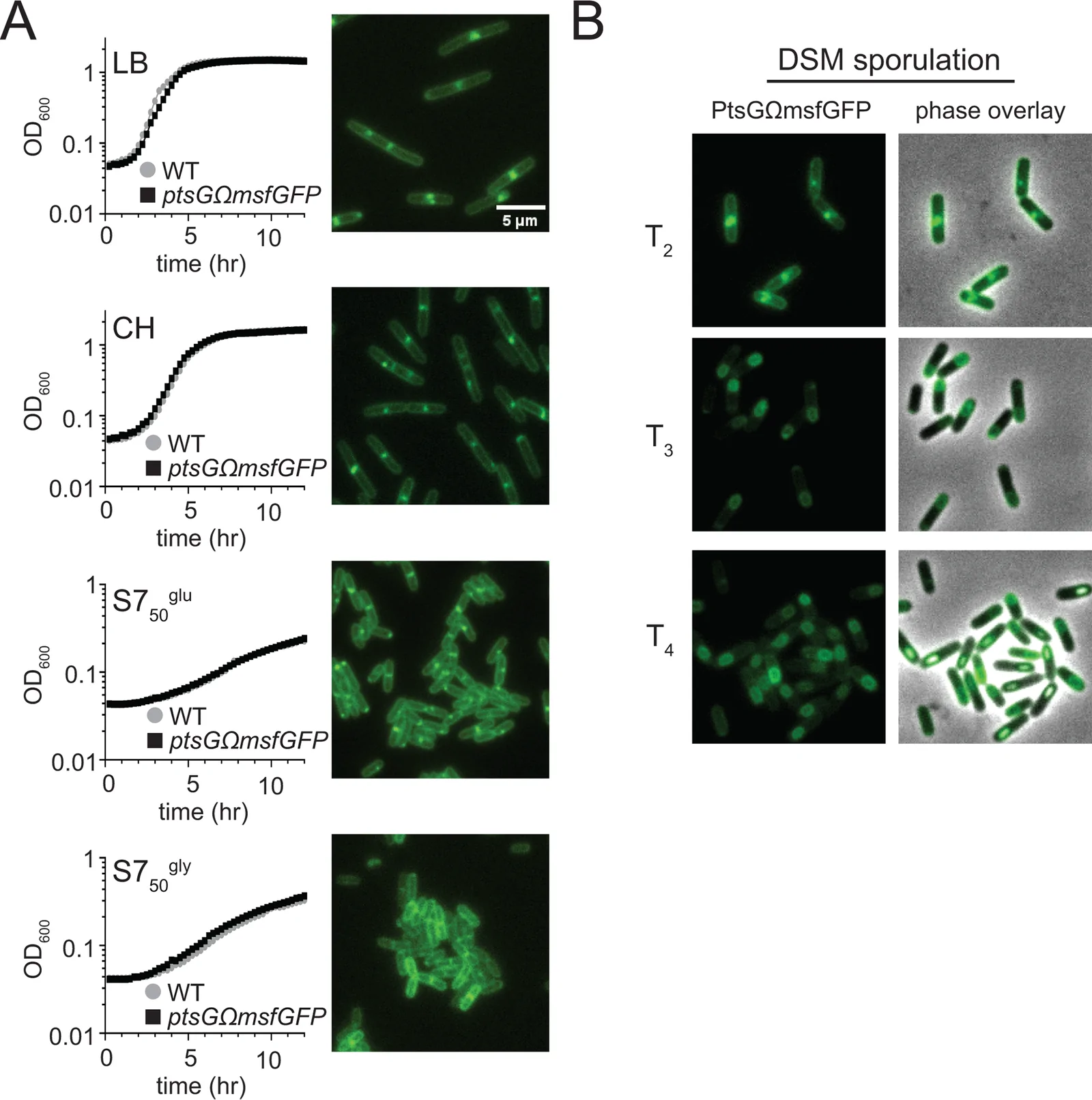
Cells expressing PtsGΩmsfGFP grow like wild type in a variety of media. (**A**) Graphs indicate growth curves conducted in a plate reader with 1 mL volumes of the indicated media type and 24-well plates. Data are plotted as optical density at 600 nm (OD_600_) over time. Black squares, *ptsGΩmsfGFP* (DB1567); gray circles, wild type (DK1042). Micrographs indicate fluorescence of PtsGΩmsfGFP (DB1567) from cells grown in the same media as indicated by the graph at the left. Scale bar is 5 μm. (**B**) Micrographs false-colored and overlaid shown under DSM sporulation indicate that PtsGΩmsfGFP can track spore formation and indicate where spores have formed within the cell body. Micrographs were taken at hours 2 (T_2_), 3 (T_3_), and 4 (T_4_) after back dilution in DSM-complete media with a 500 ms exposure time for fluorescence.

PtsG is a highly expressed membrane protein and mediates the high-affinity uptake of glucose (23, 24). As the msfGFP fusion was integrated and thus, likely, disrupted the function of the native copy of the *ptsG* gene, we tested whether the PtsGΩmsfGFP fusion exhibited any pleiotropic effects on growth. Cells expressing PtsGΩmsfGFP grew like wild type in lysogeny broth (LB, rich undefined media), casein hydrolysate broth (CH, rich defined media), and S7_50_ broth (minimal defined media) supplemented with either 1% glucose or 1% glycerol (Fig. 3A). We conclude that the expression of PtsGΩmsfGFP appeared to have little to no deleterious effects on cell growth. While we cannot account for all growth parameters, the absence of phenotype for those we tested supports the use of PtsGΩmsfGFP as a neutral indicator of membrane localization in fluorescence microscopy. Moreover, while the operon encoding PtsG is poorly expressed in minimal media, fluorescence appeared constitutive under all growth conditions tested here, perhaps because PtsG, which was inactivated by transposition, is required for autorepression (24).

To deploy PtsGΩmsfGFP as a membrane reporter in a wide variety of genetic environments, we compared fluorescence in both the NCIB 3610 ancestral strain of *B. subtilis* as well as the commonly used laboratory strain derivative PY79. Fluorescence was strong in both the ancestral and laboratory strains, supporting general utility independent of genetic background (Fig. S3A). Moreover, fluorescence microscopy is often performed either by immobilizing cells on coverslips treated with poly-L-lysine, which, while expedient, can disrupt subcellular localization in some cases, or on agarose pads, which tend to preserve delicate subcellular localization patterns. Robust membrane labeling was observed with both treatments, indicating that users could freely choose their mounting protocol depending on their protein of interest (Fig. S3B). Membrane blebs were occasionally observed and more frequent on agarose pads, something that can also occur with exogenous membrane stains. Finally, membrane fluorescence remained present after the cessation of growth and induction of sporulation, suggesting that PtsGΩmsfGFP is also useful for labeling the membrane in post-exponential cells (Fig. 3B).

To obtain higher resolution growth parameters at the individual cell level, wild-type and PtsGΩmsfGFP-expressing cells were grown in microfluidic channels. PtsGΩmsfGFP blebs were almost never observed in cells growing in the channels (Movies S1 and S2), suggesting that bleb-like aggregates observed previously are likely technical artifacts that arise during sample preparation. For each strain, cell division events were measured with the previously established definition for cytokinesis of a local 20% reduction in a constitutively expressed cytoplasmic mCherry signal (14). Wild-type cells grew with a division time of 61 ± 12 min and an average cell length of 4.2 ± 0.9 µm. Cells expressing PtsGΩmsfGFP grew with a division time of 58 ± 18 min and an average cell length of 4.3 ± 0.9 µm. We note that the division time was longer than previously reported because the current measurements were taken at 30°C, whereas previous publications grew cells at 37°C (14). Nonetheless, there was no statistically significant difference in division time or cell length between cells that expressed the PtsGΩmsfGFP fusion and cells that did not. We conclude that the presence of the PtsGΩmsfGFP fusion had no discernible effect on cell growth either in test tubes or in microfluidic devices, at least under the conditions tested.

### Endogenous membrane labeling improves time resolution of cell division

The definition of cytokinesis based on a local 20% reduction in cytoplasmic fluorescence was considered a conservative measurement of cell division because *B. subtilis* can complete septum formation before proceeding to the conditional and non-essential step of cell separation (14, 25, 26). Given the problematic combination of the narrow septum (80 nm wide) (27), the resolution limit of fluorescence microscopy, and the fact that the measurement was a loss of signal, a 20% reduction was presumed to follow the event of septum formation by an indeterminate period. To determine whether PtsGΩmsfGFP could improve the time resolution of cytokinesis, the change in PtsGΩmsfGFP and cytoplasmic mCherry fluorescence intensity was compared over the course of the cell cycle in the same cells.

**Fig 4:**
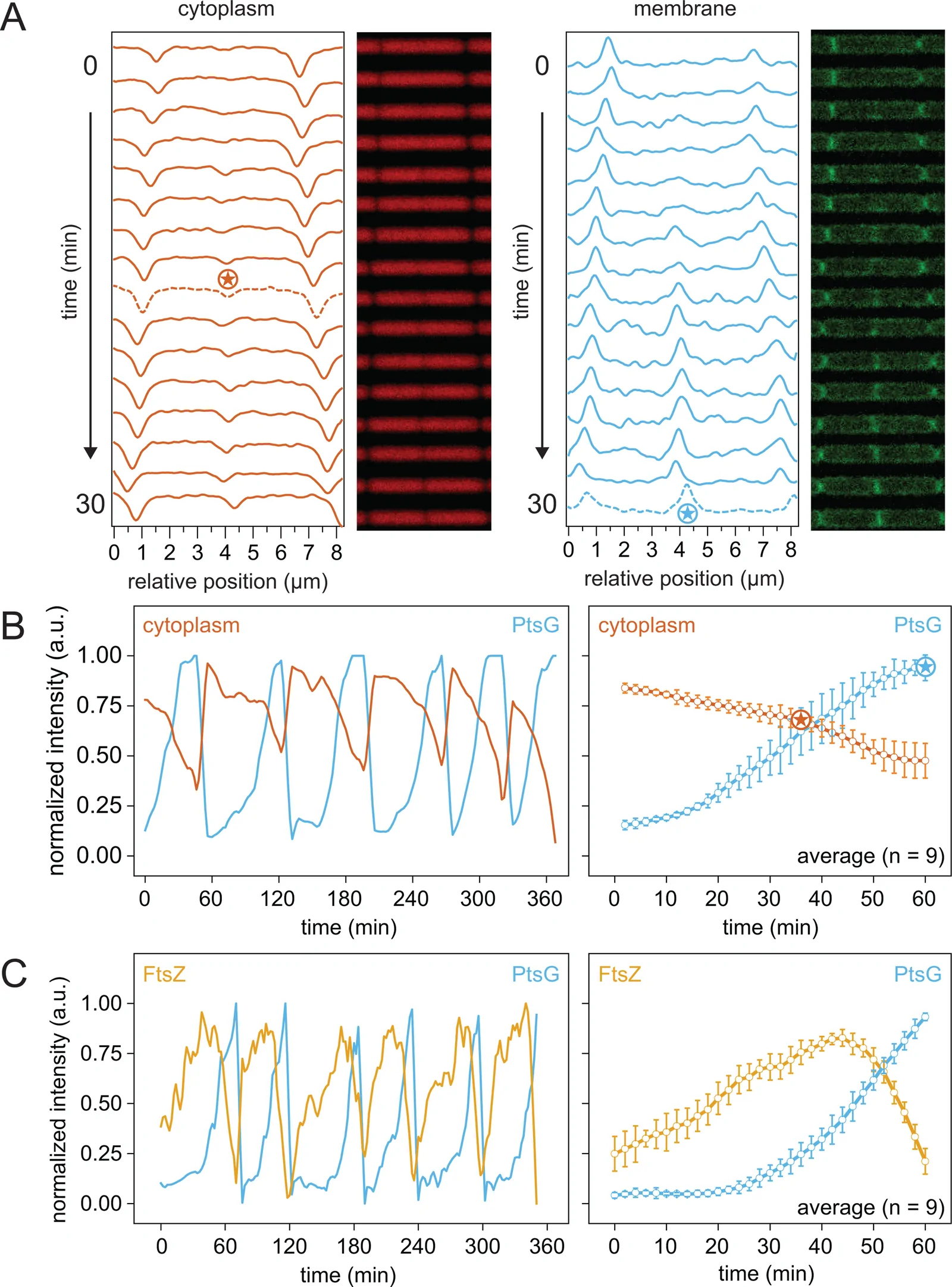
Comparison of cytoplasm labeling, membrane labeling, and mCherry-FtsZ. (**A**) Fluorescence kymograph of strain DB1756 expressing cytoplasmic mCherry (false-colored red, exposure of 200 ms, left) and PtsGΩmsfGFP (false-colored green, exposure of 200 ms, right) during growth in a microfluidic channel. Each lane represents time points of growth separated by 2-min intervals. Graphs are fluorescence intensity traces of the micrograph lane immediately to the right. Red star indicates a peak in the dotted red line where there was a local 20% reduction in cytoplasmic fluorescence intensity according to the definition of cytokinesis set previously (14). Cyan star indicates a peak in the dotted cyan line where local membrane fluorescence intensity reached a maximum to create a new definition for cytokinesis. (**B**) Left: fluorescence intensity of strain DB1756 expressing cytoplasmic mCherry (red), and the PtsGΩmsfGFP membrane label (cyan) of multiple cell division events expressed as arbitrary units (a.u.). Right: an average of the fluorescence intensity of the cytoplasmic mCherry (red) and the PtsGΩmsfGFP membrane label (cyan) of *n* = 9 division events during growth in a microfluidic channel. Red star indicates the point at which cytoplasmic fluorescence intensity decreased 20%, and cyan star indicates the point of maximum membrane intensity. Error bars are standard error. (**C**) Left: fluorescence intensity of strain DB2118 expressing mCherry-FtsZ (gold), and the PtsGΩmsfGFP membrane label (cyan) of multiple cell division events expressed as arbitrary units (a.u.) during growth in a microfluidic channel. Right, average fluorescence intensity of mCherry-FtsZ (gold) and the PtsGΩmsfGFP membrane label (cyan) of *n* = 9 division events. Error bars are the standard error of the mean. A sample time-lapse imaging series of the double labeling experiment is provided in Fig. S4.

Cytoplasmic fluorescence was integrated along the cell body with a rectangular region of interest and expressed graphically as a line profile with periodic troughs (Fig. 4A). The average cytoplasmic fluorescence intensity at division sites over multiple cell cycles exhibited a shallow negative slope (Fig. 4B, red) with low dynamic range likely due to the nature of fluorescence loss and the point spread function of fluorescence affecting neighboring bins for local analysis. PtsGΩmsfGFP expressed in the same cell, however, gradually increased in fluorescence intensity at division sites until it fully spanned the width of the septal region and reached 100% signal intensity (Fig. 4A and B, cyan star). We conclude that defining cytokinesis as maximal local fluorescence of PtsGΩmsfGFP was more facile and provided a broader dynamic range than the loss of cytoplasmic fluorescence. Moreover, we note that contrary to expectations, a local 20% reduction in mCherry cytoplasm fluorescence (Fig. 4A and B, red star) preceded the direct observation of septum formation and did so by approximately one quarter of the total cell cycle.

**Fig 5:**
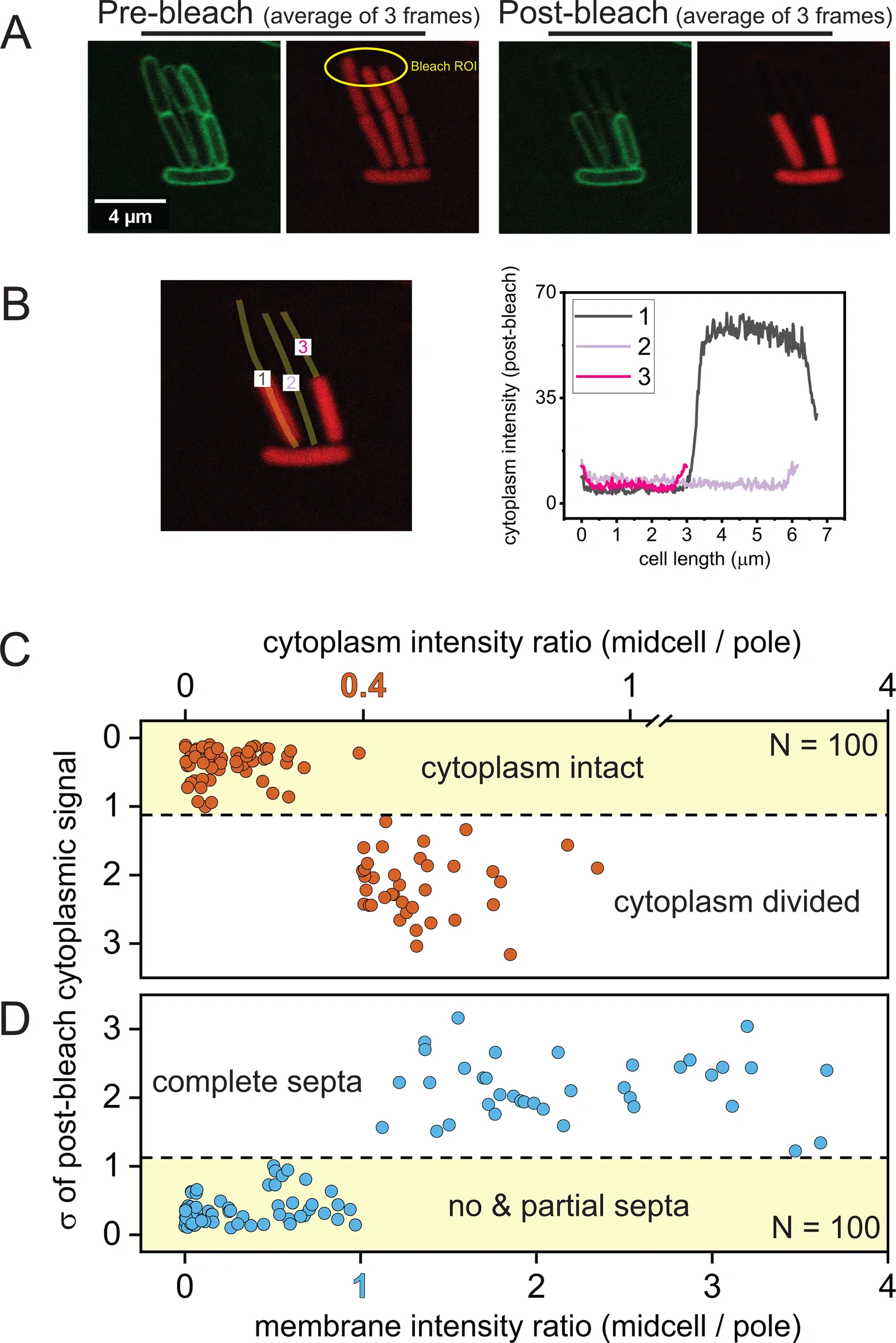
Bleach ratio by FLIP imaging indicates when cytoplasms become separate. (**A**) Fluorescence microscopy of a sample field of DB1756 cells with membranes labeled by PtsGΩmsfGFP (false-colored green) and cytoplasms labeled by mCherry (false-colored red) on 1% agarose CH pads. Images were acquired at a scan speed of 400 Hz with a pixel dwell time of 1.2 μs. The membrane signal was collected at 4% laser intensity with an excitation at 488 nm and collected at 501–547 nm. The cytoplasm signal was collected at 2% laser intensity with an excitation at 575 nm and collected at 594–651 nm. The pre-bleach and post-bleach images are an average of three frames at intervals of 2.58 s. The yellow oval represents the bleach region of interest (ROI) placed in the FRAP wizard for the bleaching iterations, and the scale bar is 4 μm. (**B**) The image is the same as the right-most panel in Fig. 5A as a representative example of data analysis. For each cell, the membrane pre-bleach series was used to place a segmented line across the cell body and was saved as an ROI to apply to the other images of the same cell. Data for *N* = 100 cells were generated with this approach. (**C and D**) Cytoplasmic intensity and membrane fluorescence were measured along each line scan prior to bleaching, and for each line scan, fluorescence at midcell was divided by fluorescence at the poles to generate cytoplasm (panel C) and membrane (panel D) intensity ratios (x-axes). The y-axis indicates the standard deviation (σ) of the post-bleach cytoplasmic signal divided by 10 (for scaling purposes). σ values below 1.125 (dotted line) were deemed to represent contiguously intact cytoplasms, whereas values above the threshold were deemed to represent cells that had been separated by cytokinesis.

To determine how the two definitions of cytokinesis relate to the separation of cell cytoplasm, fluorescence loss in photobleaching (FLIP) experiments were collected for one hundred cells. Cells were individually analyzed to determine the signal intensity at the midcell and pole for the cytoplasmic and membrane labels. An example of images used in data analysis shows the membrane fluorescence and cytoplasmic fluorescence, both prior to and after photobleaching of one cell pole (Fig. 5A). For both the membrane and cytoplasmic-based definitions, X-axis values were determined by dividing the intensity of signal at the midcell by the intensity of signal at the cell pole. The bleach ratio (Y-axis) is the standard deviation of the cytoplasmic fluorescence intensity after bleaching divided by 10 (for scaling purposes) (Fig. 5B and C). When the cytoplasmic intensity at midcell reached 0.4 (40%) of the signal at the cell pole, the cytoplasms of the two compartments shifted from being intact (yellow background) to divided (white background) (Fig. 5B). By contrast, the cytoplasms became divided when the membrane intensity at midcell reached 1 (100%) of the maximum membrane signal at the cell pole (Fig. 5C). We conclude that the cytoplasmic-based measurement of cytokinesis is precise and should be adjusted to a 40% reduction of fluorescence intensity at midcell for better accuracy. We further conclude that cytokinesis occurs when membrane fluorescence at the midcell reaches a maximum.

Previous work had indicated that the cell division protein FtsZ persisted for a period of time at the cell pole after cytokinesis (14). The parameters of FtsZ dynamics, however, were measured in the context of using a reduction in cytoplasmic fluorescence that now seemed to substantially precede cytokinesis as measured by membrane staining. To study the relationship between FtsZ-ring dynamics and membrane synthesis, a strain expressing an mCherry-FtsZ fusion protein and a PtsGΩmsfGFP fusion simultaneously was grown in microfluidic devices. FtsZ fluorescence appeared first at the future site of cell division and increased over time (Fig. 4C; Fig. S4). Indeed, FtsZ continued to accumulate at the midcell prior to the first appearance of membrane invagination presumably due to the maturation of the FtsZ ring that is needed to initiate septum construction (Fig. 4C). After septum initiation, fluorescence intensity of both signals increased until the membrane fluorescence reached approximately 50% of maximum intensity, at which point FtsZ intensity peaked and began to decrease (Fig. 4C). We conclude that FtsZ fluorescence intensity poorly correlates with that of PtsGΩmsfGFP, that peak FtsZ intensity occurs after the initiation of septum formation, and that FtsZ-ring disassembly is complete at or near the point of cytokinesis.

**Fig 6:**
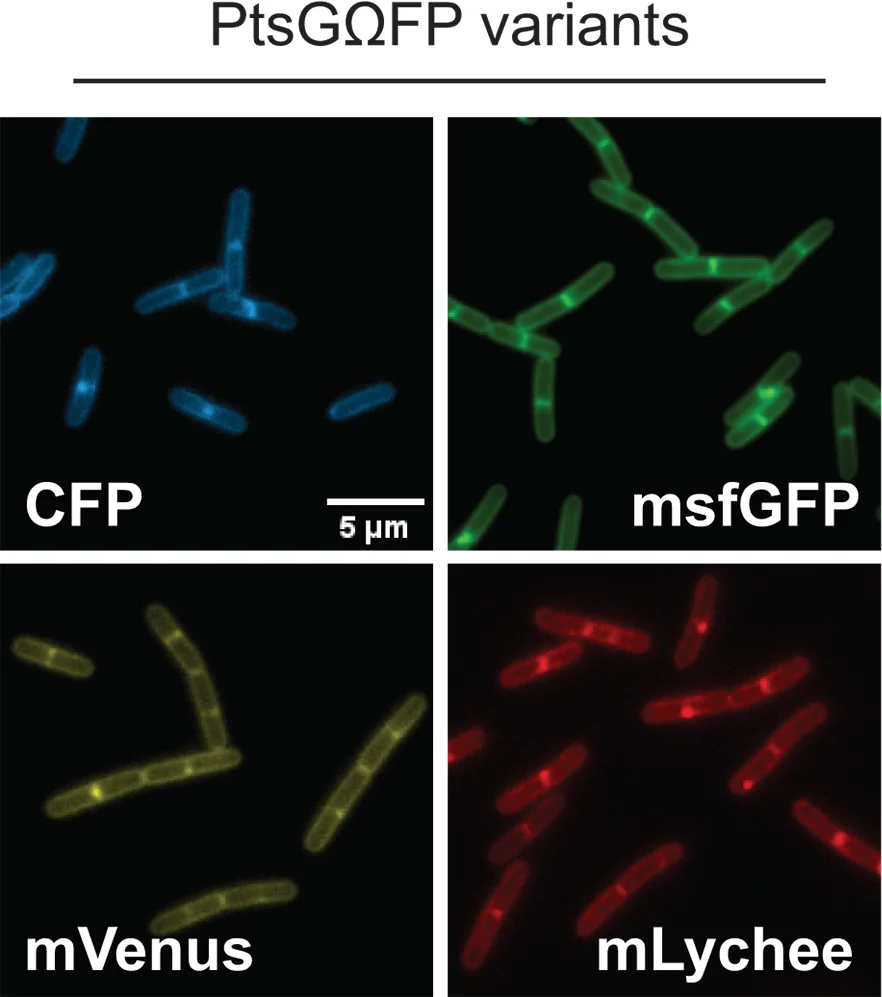
Variants of PtsG membrane labels with different fluorophore and antibiotic combinations. Fluorescence micrographs of the following strains: CFP (DB3548, exposure 400 ms), msfGFP (DB3267, exposure 500 ms), mVenus (DB3547, exposure 500 ms), and mLychee (DB3500, exposure 700 ms). Cells were imaged on 1% agarose pads, and the scale bar is 5 µm.

To maximize the potential for deployment in *B. subtilis*, four different fluorescent proteins were recombined with four different antibiotic cassettes to enhance compatibility with pre-existing cell biological constructs (Table 2 and Fig. 6).

**TABLE 2:** PtsG-fluorescent protein fusion constructs

Fusion	Antibiotic resistance	Strain^*a*^
*PtsGΩmsfGFP*	Spectinomycin	DB2645
*PtsGΩmsfGFP*	Kanamycin	DB2055
*PtsGΩmsfGFP*	Tetracycline	DB2056
*PtsGΩmsfGFP*	Chloramphenicol	DB3015
*PtsGΩCFP*	Spectinomycin	DB1854
*PtsGΩCFP*	Kanamycin	DB2058
*PtsGΩCFP*	Tetracycline	DB2059
*PtsGΩCFP*	Chloramphenicol	DB3472
*PtsGΩmVenus*	Spectinomycin	DB1855
*PtsGΩmVenus*	Kanamycin	DB2061
*PtsGΩmVenus*	Tetracycline	DB2062
*PtsGΩmVenus*	Chloramphenicol	DB3471
*PtsGΩmLychee*	Spectinomycin	DB3499
*PtsGΩmLychee*	Kanamycin	DB3549
*PtsGΩmLychee*	Tetracycline	DB3550
*PtsGΩmLychee*	Chloramphenicol	DB3551

^a^ All strains are in the PY79 laboratory strain genetic background.

## DISCUSSION

The use of fluorescence microscopy for the analysis of protein function is common in *B. subtilis*, and membrane stains, such as FM4-64 or TMA-DPH, are often used to provide context for protein subcellular localization. Fluorescent dyes are relatively inexpensive and easy to add for a single time point analysis, but the staining procedure can be difficult to optimize, and excess dye incorporation may cause membrane perturbations. The challenge of consistent staining is exacerbated in experiments involving time-dependent observation. We sought to replace dye addition by the expression of a fluorescent protein that localizes to the membrane and is continually produced by *B. subtilis*. Here, we use an unbiased forward genetic approach to create random fluorescent protein fusions and screen for those that enrich in the membrane. We show that fluorescent protein fusions to PtsG provide for constitutive and intense membrane fluorescence suitable for both instantaneous and time-dependent observation over a variety of conditions without conferring a defect in growth.

PtsG is the transmembrane component of the high-affinity phosphoenolpyruvate-dependent PTS transporter for glucose, and the native-site fusion used here is almost certainly non-functional for glucose import. We were initially concerned that the fusion might cause growth defects, but cells containing the PtsGΩmsfGFP fusion grew like wild type even when grown in minimal media with glucose as the sole carbon and energy source, presumably due to the presence of other glucose uptake systems. We were also concerned that PtsGΩmsfGFP fluorescence might be conditional, as *ptsG* gene expression has been shown to vary (24), but the fluorescence intensity of PtsGΩmsfGFP seemed constant during both growth and sporulation. While native site integration restricts use to *B. subtilis* strains with high sequence similarity surrounding the *ptsG* locus, we note that PtsG is highly conserved across bacteria, and we show that it would be a suitable candidate for generating constitutive membrane fluorescence in other organisms.

One application of PtsGΩmsfGFP is dynamic membrane labeling during microfluidic growth, which demonstrates that the point at which septal membrane fluorescence reaches a maximum serves as a robust, albeit indirect, definition of cytokinesis that is also supported by FLIP microscopy (Fig. 5D). By comparison, we find that cytokinesis defined by a local 20% reduction in fluorescence of a cytoplasmically expressed protein precedes membrane transection by approximately one quarter of the cell cycle. Tracking the cytoplasm was precise, and changing to a local 40% reduction in fluorescence would make it accurate to the cell cycle and equivalent to the membrane-based definition (Fig. 5C). Simultaneous observation with fluorescent FtsZ fusion indicates that FtsZ appears at the new division site before an increase in membrane fluorescence. The FtsZ focus continues to intensify during membrane constriction to a maximum, after which disassembly is rapid to extinction that roughly coincides with the formation of a membrane transect. Thus, we infer that the rate of FtsZ recruitment may be constant but that the rate of disassembly either gradually increases during constriction or initiates after a checkpoint. Whatever the case, direct observation of membrane constriction clarified the order of events during division, and contrary to our conclusions using the cytoplasmic definition of cytokinesis (14), FtsZ does not appreciably dwell at the cell poles after division is complete, at least in the wild type. We conclude that PtsGΩmsfGFP is a robust tool for fluorescent labeling of the membrane and can be modified as necessary to meet the needs of the user.

Fluorescent fusions to PtsG recombining four different fluorophores and four different antibiotic resistance cassettes will be donated to the Bacillus Genetic Stock Center (BGSC, The Ohio State University), and the sequence of the PtsGΩmsfGFP is included in the supplemental materials to aid future modifications.

## MATERIALS AND METHODS

### Strains and growth conditions

*B. subtilis* strains were grown in LB (10 g tryptone, 5 g yeast extract, and 5 g NaCl per L) or on LB plates fortified with 1.5% Bacto agar at 37°C. When appropriate, antibiotics were included at the following concentrations: 10 µg/mL tetracycline, 5 µg/mL chloramphenicol, 5 µg/mL kanamycin, 100 µg/mL spectinomycin, and 1 µg/mL erythromycin plus 25 µg/mL lincomycin (mls). Isopropyl β-D-thiogalactopyranoside (IPTG, Sigma) was added to the medium at the indicated concentration when appropriate. As needed, cells were grown in the rich defined media casein hydrolysate (CH), prepared as described previously (28, 29).

S7_50_ media were prepared in 100 mL aliquots by mixing 10 mL 10× S7_50_ salts (recipe below), 1 mL 100× S7_50_ metals (recipe below), 2 mL 1 M glutamate, and 2 mL of 50% glucose or glycerol together with 82 mL dH_2_O. All components (except for the dH_2_O) were filter-sterilized.

The 10× S7_50_ salts were made in 1 L aliquots: 104.7 g MOPS (free acid), 13.2 g ammonium sulfate (NH_4_)_2_SO_4_, and 6.8 g potassium phosphate monobasic KH_2_PO_4_ were added and buffered to pH 7 with 1 M KOH in a volume of 400 mL of ddH_2_O, then made up to 1 L with ddH_2_O. Media were filter-sterilized into 100 mL aliquots, covered with foil, and stored at 4°C. If yellowed, the solution is no longer usable. The 100× S7_50_ metals have final concentrations of 0.2 M MgCl_2_, 70 mM CaCl_2_, 5 mM MnCl_2_, 0.1 mM ZnCl_2_, 2 µg/mL thiamine-HCl, 2 mM HCl, and 0.5 mM FeCl_3_. Fe was added last to prevent precipitation. The solution was filter-sterilized and stored foil-wrapped in 10 mL aliquots at 4°C.

DSM-incomplete broth was prepared as a 1 L bulk and subsequently dispensed as 100 mL aliquots. The 1 L DSM-incomplete broth was prepared by mixing 8 g of Difco Nutrient Broth, 10 mL of 100 mM MgSO_4_, 10 mL of 10% KCl, 0.5 mL of 1N NaOH, and 1 L of dH_2_O. The 100 mL aliquots are then autoclaved for 30 min and stored at benchtop conditions until ready for use. Once ready for use, add 100 µL of 1 M Ca(NO_3_)_2_, 100 µL of MnCl_2_, and 100 µL of 1 mM FeSO_4_ into a 100 mL DSM-incomplete broth aliquot to make 100 mL of DSMcomplete broth.

Casein-Hydrolase media (CH media) were prepared in separate parts and subsequently dispensed as 100 mL aliquots with the following composition: 94 mL CH I+II, 5 mL CH III, 200 μL CH IV, and 1 mL CH V. CH I+II was prepared as a 2 L bulk by mixing 20 g of casein hydrolysate (dot scientific), 9.4 g of L-glutamate (monohydrate), 3.2 g of L-asparagine (monohydrate), 2.5 g of L-alanine, 2.72 g of KH_2_PO_4_ (potassium phosphate monobasic anhydrous), 2.68 g of NH_4_Cl (ammonium chloride), 1 mL of 2,000× stock Na_2_SO_4_ (sodium sulfate) solution, 1 mL of 2,000× stock NH_4_NO_3_ (ammonium nitrate) solution, 1 mL of 2,000× stock FeCl_3_∙6H_2_O (ferric chloride 6-hydrate) solution, and 2 L of dH_2_O. The pH of CH I+II was adjusted to 7.0 with 1N NaOH. CH III was prepared as a 1 L bulk by mixing 0.66 g of CaCl_2_∙2H_2_O (calcium chloride dihydrate), 2.47 g of MgSO_4_∙7H_2_O (magnesium sulfate heptahydrate), and 1 L of dH_2_O. CH IV was prepared as a 1 L bulk by mixing 1.67 g of MnSO_4_∙H_2_O (manganese sulfate monohydrate) into 1 L of MilliQ-treated water. CH V was prepared as a 100 mL bulk by mixing 0.2 g of L-tryptophan and 100 mL of dH_2_O. CH I+II, CH III, and CH IV were all autoclaved for 30-min liquid cycles. CH V was sterilized with a 0.22-μm screw cap filter (28, 29).

### Growth curve

Cells were grown in 3 mL of the desired media, rolling at 37°C until turbid. Cultures were then back-diluted to a starting OD_600_ of 0.01. On a 24-well plate, 500 µL was transferred into the wells in triplicate and then placed without the lid into the plate reader (Agilent BioTek Synergy H1 microplate reader). The plate reader was set to double orbital shaking at a high speed at 37°C for 18 h, with OD_600_ measurements taken every 15 min with Gen5 Microplate Reader and Imager Software Version 3.11. Data were collected the next day and graphed with GraphPad Prism 10 software.

### Preparation of cells for microscopy

Cells were grown in LB broth at 37°C until log phase (OD_600_ 0.5–0.9). A total of 1 mL of cells was then pelleted. Cells were resuspended in 30 µL 5 µg/mL FM4-64 (Molecular Probes) and incubated at room temperature for 3 min to stain the membrane before being pelleted again. Cells were then washed with 1 mL 1× PBS and pelleted again. All samples were resuspended in 30 µL 1× PBS, then observed by spotting 4 µL of this suspension on a 1% agarose pad made with water. CH media can sustain growth while maintaining low background fluorescence during imaging.

For sporulation, cells were grown in 2 mL of DSMcomplete broth for 1–2 h at 37°C and then back-diluted with 25 mL of DSMcomplete to an OD_600_ of 0.01 at 37°C; 1 mL of the culture was collected every hour after the end of exponential phase to observe cell sporulation. All samples were centrifuged at 8,000 × *g* for 1 min, and the supernatant was removed. Samples were then resuspended in 30 µL of dH_2_O and imaged.

### Microscopy

Fluorescence microscopy for still images was performed with a Nikon 80i microscope along with a phase contrast objective Nikon Plan Apo 100× and an Excite 120 metal halide lamp. FM4-64 fluorescence signals were visualized with a C-FL HYQ Texas Red filter cube (excitation filter 532–587 nm, barrier filter 590 nm), and a 500 ms exposure time was used. Monomeric superfolder green fluorescent protein (msfGFP) was visualized with a C-FL HYQ FITC filter cube (fluorescein isothiocyanate [FITC]; excitation filter, 460–500 nm; and bandpass filter, 515–550 nm), and a range of 500–700 ms exposure times was used. Yellow fluorescent protein (YFP) was visualized with a C_FL HYQ YFP filter cube (excitation filter, 490–510 nm, and barrier filter, 515–550 nm) and an exposure time of 500 ms. TMA-DPH fluorescent signal was visualized with a UB-2E/C DAPI filter cube (excitation filter, 340–380 nm, and bandpass filter, 435–485 nm) and an exposure time of 500 ms. Images were captured with a Photometrics Coolsnap HQ2 camera in black and white, false-colored, and superimposed with NIS Elements Image software.

Fluorescence microscopy was also performed with a Nikon Ti2 Eclipse along with a phase contrast objective Nikon Plan Apo 100× oil-immersion objective and Lumencor Spectra light. msfGFP was excited with 474/27 nm and collected at 515/30 nm, with an exposure time of 500 ms. CFP was excited with 438/24 nm and collected at 483/32 nm, with an exposure time of 400 ms. mVenus was excited with 509/22 nm and collected at 543/22 nm, with an exposure time of 500 ms. mLychee signals were excited with 554/23 nm and collected at 595/31 nm, with an exposure time of 700 ms. Semrock Triple Band Pinkel multiband and Quad band Sedat multiband filter sets were coupled with single band emission filters for generic fluorophore spectra. Images were captured with a Prime 95B Photometrics camera engine.

### Strain construction

Strain constructs were generated either by direct transformation into PY79 and transduced into 3610 by the generalized transducing phage SPP1 (30) or by direct transformation into the competent 3610 background strain DK1042 (31). All strains used in this study are listed in Table 3. All plasmids used in this study are listed in Table S1. All primers used in this study are listed in Table S2.

### mCherry-FtsZ

To generate pLL4, the promoter region of *ftsA* was amplified from DK1042 chromosomal DNA with primers 7611/7606 and digested with HindIII/NheI, and the *ftsZ* gene was amplified from DK1042 chromosomal DNA with primers 7607/7608 and digested with NheI/BamHI. The two fragments were cloned simultaneously into the HindIII/BamHI sites of pWX146 containing a polylinker and erythromycin resistance cassette between two arms of the *yuxG* gene (generous gift of Dr. Xindan Wang, Indiana University). Plasmid pWX146 was then transformed into DK1042 to generate strain DK9790.

**TABLE 3:** Strains

Strain	Genotype
DK1042	*comI^Q12L^* (ancestral background)
PY79	*sfp^0^ swrA^FS^* (laboratory strain)
DB1396	[PY79] *ptsG^late^ΩTnFLXmsfgfp spec*
DB1401	*comI^Q12L^ ptsG^late^ΩTnFLXmsfgfp spec*
DB1567	*comI^Q12L^ ptsG^early^ΩTnFLXmsfgfp spec*
DB1701	*comI^Q12L^ ccpAΩTnFLXmsfgfp spec*
DB1756	*ΔepsH amyE::P_hyspank_-mcherry kan ptsGΩTnFLXmsfgfp spec*
DB1847	*comI^Q12L^ manPΩTnFLXmsfgfp spec*
DB1854	[PY79] *ptsGΩTnFLXcfp spec*
DB1855	[PY79] *ptsGΩTnFLXmvenus spec*
DB1864	*comI^Q12L^ ptsGΩTnFLXcfp spec*
DB1865	*comI^Q12L^ ptsGΩTnFLXmvenus spec*
DB1948	*comI^Q12L^ yybNΩTnFLXmsfgfp spec*
DB2055	[PY79] *ptsGΩTnFLXmsfgfp kan*
DB2056	[PY79] *ptsGΩTnFLXmsfgfp tet*
DB2058	[PY79] *ptsGΩTnFLXcfp kan*
DB2059	[PY79] *ptsGΩTnFLXcfp tet*
DB2061	[PY79] *ptsGΩTnFLXmvenus kan*
DB2062	[PY79] *ptsGΩTnFLXmvenus tet*
DB2118	*ΔepsH yuxG::P_ftsA_-mcherry-ftsZ mls ptsGΩTnFLXmsfgfp kan*
DB2136	*comI^Q12L^ yknWΩTnFLXmsfgfp spec*
DB2137	*comI^Q12L^ ywdKΩTnFLXmsfgfp spec*
DB2138	*comI^Q12L^ glySΩTnFLXmsfgfp spec*
DB2140	*comI^Q12L^ msmXΩTnFLXmsfgfp spec*
DB2141	*comI^Q12L^ motAΩTnFLXmsfgfp spec*
DB2142	*comI^Q12L^ guaBΩTnFLXmsfgfp spec*
DB2372	*comI^Q12L^ yidC1ΩTnFLXmsfgfp spec*
DB2645	[PY79] *ptsGΩTnFLXmsfgfp spec*
DB3015	[PY79] *ptsGΩTnFLXmsfgfp cat*
DB3471	[PY79] *ptsGΩTnFLXmvenus cat*
DB3472	[PY79] *ptsGΩTnFLXcfp cat*
DB3499	[PY79] *ptsGΩTnFLXmlychee spec*
DB3549	[PY79] *ptsGΩTnFLXmlychee kan*
DB3550	[PY79] *ptsGΩTnFLXmlychee tet*
DB3551	[PY79] *ptsGΩTnFLXmlychee cat*
DB3267	*comI*^*Q12L*^ *ptsGΩTnFLXcfp tet*
DB3500	*comI*^*Q12L*^ *ptsGΩTnFLXmsfgfp cat*
DB3547	*comI*^*Q12L*^ *ptsGΩTnFLXmvenus kan*
DB3548	*comI*^*Q12L*^ *ptsGΩTnFLXmlychee spec*
DK9790	*yuxG::P_ftsA_-ftsZ mls*

To generate an mCherry fusion to FtsZ, the region upstream of *yuxG,* including the promoter region of *ftsA,* was amplified from DK9790 chromosomal DNA with primers 7758/7759, the ftsZ gene, erythromycin resistance cassette, and the region downstream of *yuxG* was amplified from DK9790 chromosomal DNA with primers 7762/7763, and the mCherry gene was amplified from pDR201 with primers 7760/7761 (generous gift of Dr. David Rudner, Harvard Medical School). The three amplicons were stitched together with isothermal assembly (32) and transformed into DK1042.

### Fluorophore replacements

To generate the PtsG-spec alleles containing different fluorophores such as cyan fluorescent protein (CFP), mVenus, and mLychee, each cassette was separately PCR-amplified from either pDR200 (*cfp*), pFK116 (*mvenus*), or pDP679 (*mlychee*). The primer pairs for each plasmid were 8498/8499 for *cfp*, 8488/8489 for *mvenus,* and 9060/9061 for *mlychee* (33). Two fragments, one containing the 5′ end of the *ptsGΩpTnFLXmsfgfp* insertion plus the region upstream, and the other containing the 3′ end of the *ptsGΩpTnFLXmsfgfp* insertion plus the region downstream, were separately PCR-amplified from DB1567 chromosomal DNA using primers specific to each fluorophore: *cfp* (8486/8490 and 8491/8487), *mvenus* (8486/8496 and 8491/8487), and *mlychee* (8486/8886 and 9062/8487). Each of the two chromosomally amplified fragments was mixed with a fragment encoding the cognate fluorophore cassette and ligated by Gibson isothermal assembly (32). The resulting fragments were transformed into PY79 selecting spectinomycin resistance. The constructs were confirmed by amplifying the region using primer pair 8486/8487 and sequencing the amplicon.

### Antibiotic replacements

To generate the PtsGΩmsfGFP alleles containing kanamycin (kan) and tetracycline (tet) resistance cassettes, each cassette was separately amplified from either pDG780 (kan) or pDG1515 (tet) (34) with primer pair 3250/3251. Two other fragments containing the 5′ *ptsGΩpTnFLXmsfgfp* insertion plus the region upstream and a fragment containing the 3′ end of the *ptsGΩpTnFLXmsfgfp* insertion plus the region downstream were separately PCR amplified from DB1567 chromosomal DNA with primer pairs 8486/8516 and 8487/8517. Each of the two chromosomally amplified fragments was mixed with a fragment encoding an antibiotic cassette and ligated by Gibson isothermal assembly (32). The resulting fragments were transformed into PY79, with transformants selected for kanamycin and tetracycline resistance, respectively. The constructs were confirmed by amplifying the region with primer pair 8486/8487 and sequencing the amplicon.

To generate the PtsGΩmsfGFP allele containing chloramphenicol (cat) resistance cassettes, the cassette was amplified from pAC225 (generous gift of Arnaud Chastanet and Rich Losick, Harvard University) with primer pair 3250/3251. Two other fragments containing the 5′ *ptsGΩpTnFLXmsfgfp* insertion plus the region upstream and a fragment containing the 3′ end of the *ptsGΩpTnFLXmsfgfp* insertion plus the region downstream were separately PCR-amplified from DB1567 chromosomal DNA with primer pairs 8486/8884 and 8487/8885. The three fragments were mixed and ligated by Gibson isothermal assembly (32). The resulting fragment was transformed into PY79, selecting for chloramphenicol resistance, and confirmed by amplifying the region with primer pair 8486/8487 and sequencing the amplicon.

To generate chloramphenicol-resistant variant fluorophores fused to PtsG, the gene for chloramphenicol (cat) resistance was amplified from pAC225 with primer pair 3250/3251. Next, the upstream and downstream arms were amplified as follows: *ptsGΩpTnFLXcfp* was amplified from DB1854 chromosomal DNA with primers 8486/9043 and 8487/8885, *ptsGΩpTnFLXmvenus* was amplified from DB1855 with primers 8486/9042 and 8487/8885, and *ptsGΩpTnFLXmlychee* was amplified from DB3499 with primers 8486/9087 and 8487/8885. The cognate arms were mixed with the chloramphenicol resistance cassette amplicon and ligated by Gibson isothermal assembly (32). The resulting fragment was transformed into PY79, selecting for chloramphenicol resistance, and confirmed by amplifying the region with primer pair 8486/8487 and sequencing the amplicon.

### iPCR and sequencing products

An iPCR product containing the transposed gene was amplified from *B. subtilis* chromosomal DNA of each mutated strain with the primers 6212/6420. The iPCR products were then sent for sequencing using primer 6224.

### Microfluidics

#### Materials

Materials used to fabricate microfluidics devices included glass slides (50 mm by 75 mm) from Corning, Inc.; poly(dimethylsiloxane) (PDMS, Sylgard 184) from Dow Corning, Inc.; No. 1.5 Gold Seal coverslips (48 mm by 60 mm) from VWR International, LLC; SU-8 2010 photoresist and SU-8 developer from Kayaku Advanced Materials, Inc.; titanium di-isopropoxide bis(2,4-pentanedionate) from Gelest, Inc.; and all other chemicals from Sigma-Aldrich Co.

#### Device fabrication and operation

The microfluidic devices consisted of three layers, including a glass cover slide (bottom), a ~100-µm thick PDMS fluid layer (middle), and a 3-mm thick PDMS control layer (top). The PDMS layers were fabricated through a mold-replica process (2, 14). The SU-8 mold for the fluid layer was formed in two steps: electron beam lithography (FEI Quanta 600F scanning electron microscope equipped with a JC Nabity Nanometer Pattern Generation System) generated an array of approximately 1,000 microchannels (1.0 µm deep and 0.5–1.0 µm wide) followed by photolithography (OAI 200 Mask Aligner) to pattern microchannels (20 µm deep and 100 µm wide) positioned perpendicularly to the microchannel array. The microchannels in the control layer were generated in a single photolithographic step.

PDMS in a 10:1 ratio of polymer to cross-linker was spin-coated onto the fluid-layer mold and poured onto the control-layer mold and degassed. The PDMS for the fluid layer was partially cured at 75°C for 7 min, whereas the PDMS for the control layer was fully cured at 75°C for 1 h. The PDMS control replicas were removed from the molds, and a biopsy punch was used to bore holes through the control layer. The fluid and control layers were aligned and fully cured at 75°C for 4 h. The side of the fluid layer with the microchannel array was plasma cleaned (Harrick PDC-001) and bonded to the glass coverslip. The assembled device was placed in a holder that was pneumatically connected to a control box to actuate on-device valves and pumps to control movement of media and cells.

Microchannels were filled with 0.1% bovine serum albumin (BSA) in media, and valves were checked for functionality. Media were fed through the device by gravity and directed to various locations on the device by opening and closing the valves with vacuum and pressure, respectively. Cells were loaded and trapped in the microchannel array by applying pressure above the microchannel array through the control layer. Cells were allowed to grow and acclimate for approximately 2 h before the analysis began.

#### Time-lapse image acquisition

Fluorescence images were collected on a Nikon Ti2 microscope with a Hamamatsu ORCA-Fusion Digital CMOS camera, 100× Plan Apo oil-immersion objective (1.45 NA), and Lumencor spectra light engine. mCherry was excited with 540–565 nm, and emission was collected at 580–610 nm with an exposure time of 200 ms. msfGFP was excited with 460–500 nm, and emission was collected at 515–550 nm with an exposure time of 200–300 ms. The objective warmer (Okolab) was set at 30°C for all experiments. Nikon NIS-Elements was used for multi-color image acquisition at 2-min intervals at 12 locations within the microchannel array. Denoise.ai mode was used to align images due to stage jitter.

#### Data analysis

Image files in ND2 format were parsed into 12 separate files based on the 12 locations imaged within the microchannel array. With Fiji software (ImageJ), horizontal positions of the images were adjusted to 0° to track cell position over time, and backgrounds were subtracted from each set of images (35). Kymographs of individual channels containing viable cells were generated from images taken at 2-min intervals, and localization of PtsGΩmsfGFP (membrane), mCherry-FtsZ, and mCherry (cytoplasm) was manually tracked across the kymographs. To track cell division, cell length, and FtsZ ring dynamics, a rectangular region of interest for each cell was used to integrate the fluorescence signals and generate single-line profiles parallel to the cell length. For the cytoplasmic dye (mCherry), a 20% local decrease between adjacent cells was used as the threshold to indicate cytokinesis, whereas for the membrane label (PtsGΩmsfGFP), a maximum in the fluorescence signal was used as the threshold for cytokinesis. Progeny of the mother cells were followed by continuous tracking of fluorescence signals.

#### Fluorescence-activated cell sorting

Sorting was done by the Indiana University Bloomington (IUB) Flow Cytometry Core Facility (FCCF, RRID: SCR_024398). Samples were grown in LB broth overnight at room temperature rolling. The next day, 100 µL of culture was back-diluted into 3 mL of LB broth and rolled at 37°C for 1 h or until the OD_600_ reached 0.3; 1 mL of cells was collected in a 2 mL Eppendorf tube and centrifuged at 14,000 RPM for 2 min. The supernatant was removed, and the pellet was resuspended in 1 mL of 1× PBS buffer. Samples were then taken to the IUB FCCF for sorting based on the scatter and fluorescence properties of the cells.

The instrument used for the sort was a 5-laser SORP FACSAriaIIu with FACSDiva software version 9.4 (BD Biosciences, San Jose, CA). WT DK1042 cells, which have no inherent fluorescence, were used to set the boundary between msfGFP-negative and -positive cells. Cells were sorted at room temperature with an 85-μm nozzle set at 35 psi in a four-way purity sort mode. A 488 nm 100 mW laser was used for scatter and msfGFP detection. The parameters used for the sorting were FSC-PMT at 300 V, SSC at 269 V, and B530/30 at 650 V, all on log-scaled axes. To exclude instrument noise, debris, and other non-bacterial particles, the threshold was set at 200 on SSC; 3,800 fluorescent cells were collected in LB broth and grown up rolling overnight at 37°C. Cells were spun down in glycerol and stored at –80°C for further analysis.

#### Bleach ratio by fluorescence loss in photobleaching

Cells were grown overnight on LB kanamycin plates at 37°C, and single colonies were inoculated in 3 mL of CH media at 37°C until lag phase (OD_600_ 0.2–0.4); 1 mL of liquid culture was collected and centrifuged, and supernatant was discarded. The pellets were resuspended in 1 mL of 1× PBS and centrifuged, and supernatant was discarded again. The pellets were resuspended in 20–30 μL of CH media; 4 μL of the resuspended pellet was pipetted onto a 1% agarose CH media pad.

Image acquisition for the bleach ratio by fluorescence loss in photobleaching experiments was done on an inverted Leica SP8 scanning confocal microscope equipped with an HC PL Apo 63× oil immersion objective, 405 nm laser, and white light lasers (WLLs). For the bleach ratio experiments, image acquisition was done with the LAS-X software and the FRAP wizard. The WLL was set to 4% intensity at 488 nm for msfGFP excitation, and emission was collected at 501–547 nm. For mCherry, the WLL was set to 2% intensity at 575 nm for excitation, and emission was collected at 594–651 nm. For bleaching, the 405 nm laser was employed at 80% intensity. Images were collected with a pixel dwell time of 1.2 μs and a scan speed of 400 Hz. The FRAP wizard settings were three pre-bleach iterations at 2.580 s, 18 bleach iterations with an interval of 2.580 s, and three post-bleach iterations at 2.580 s. The two detectors used were HyD and a PMT.

Data collection for line plot profiles and averaging images was completed in Fiji, and processing of the data was done in OriginPro (35). Images were extracted from the original LIF file and were separated based on the sequence of imaging and the excitation channel. The pre-bleach and post-bleach series were averaged for a single location. The membrane (msfGFP) channel for the pre-bleach series was used to create segmented lines in Fiji and extract line plot profiles for all pre-bleach and post-bleach series (35). Three types of line plot profile data were collected, and ROIs were saved for each cell in the images: membrane pre-bleach, cytoplasm pre-bleach, and cytoplasm post-bleach. The membrane and cytoplasm pre-bleach line plot profiles used the peak intensity for the midcell and pole to normalize the septum and cytokinetic progression for each cell. The cytoplasm post-bleach line plot profile data were processed to obtain standard deviation values for each cell. Post-bleach cytoplasmic plot profile data were either completely flat (low standard deviation/fully bleached) or had a stepwise-like trend, with half the signal being high and half the signal being low (high standard deviation/half bleached). Standard deviations were divided by 10 for scaling on the graph. This method of data collection was similar to the microfluidic data analysis to keep consistency and allow the results to be comparable in % septum/cytokinesis progression.

## SUPPLEMENTAL MATERIAL

Supplemental material aem.01126-26-s0001.pdf Tables S1 and S2, PtsGΩmsfGFP sequence, and Fig. S1 to S4.

Movie S1 aem.01126-26-s0002.mp4 Cells grown in a microfluidic channel expressing PtsGΩmsfGFP and mCherry-FtsZ.

Movie S2 aem.01126-26-s0003.mp4 Cells grown in a microfluidic channel expressing PtsGΩmsfGFP and mCherry-FtsZ, high zoom.

ASM does not own the copyrights to Supplemental Material that may be linked to, or accessed through, an article. The authors have granted ASM a non-exclusive, world-wide license to publish the Supplemental Material files. Please contact the corresponding author directly for reuse.
