# Supplementary material for: Constitutive, endogenous, fluorescent membrane reporters for dynamic cell cycle analysis in Bacillus subtilis
Source: Appl Environ Microbiol. 2026 Aug 14;92(9):e01126-26. doi: 10.1128/aem.01126-26 (PMC13599486; doi:10.1128/aem.01126-26)
Supplement: Supplementary file 1 [file aem.01126-26-s0001.pdf]

SUPPLEMENTAL TABLES

Table S1: Plasmids

Plasmid	Genotype
pAC225	*cat amp*
pDG1515	*tet amp*
pDG780	*kan amp*
pDP678	*amyE::*$P_{hyspank}$*-mlychee spec amp*
pDR200	*cfp kan amp*
pFK116	*amyE::*$P_{hyspank}$*-mvenus kan amp*
pFK162	*pTnFLXmsfgfp* $spec^{Bs}$ ori^{TsBs} $spec^{Ec}$

Table S2: Primers

Primer	Sequence
3250	ACGACTCACTATAGGGCGAATTG
3251	CTCACTAAAGGGAACAAAAGCTGG
6212	CAAAAGCTGGGTACCGGG
6224	GGCTCTTTAAGAATTCTATCAG
6420	GGCTCTTTAAGAATTCTATCAGGCACTTCCGGACTCAATGGTGAGGGGAGACCGGGGAC
8486	CAGTCACTATATCCTGAGGAGTACC
8487	CGTGCCTGTAGCAGCTTTCGTTC
8488	CTGATAAGTCCCCGGTCTATGGTCTCTAAAGGAGAAGAATTG
8489	CGCCCTATAGTGAGTCGTGGGTTACTTGTAAAGTTCATCCATACCGAG
8490	CAATTCTTCTCCTTTAGAGACCATAGACCGGGGACTTATCAG
8491	CTCGGTATGGATGAACTTTACAAGTAACCCACGACTCACTATAGGGCG
8498	CTGATAAGTCCCCGGTCTATGGTTTCAAAAGGCGAAGAACTG
8496	CAGTTCTTCGCCTTTTGAAACCATAGACCGGGGACTTATCAG
8497	CTTGGCATGGACGAACTTTATAAGTAACCCACGACTCACTATAGGGCG
8499	CGCCCTATAGTGAGTCGTGGGTTACTTATAAAGTTCGTCCATGCCAAG
8516	GGTGGAGCTCCAATTCGCCCTATAGTGAGTCGT
8517	GGCCCGGTACCAGCTTTTGTTCCCTTTAGTGAG
8884	CCAGCTTTTGTTCCCTTTAGTGAGGGGTTATTTATATAACTCGTCCATCCC
8885	CAATTCGCCCTATAGTGAGTCGTGGGAGACCGGGGACTTATCAGCCAACCTGT
8886	GCTAGCGCCAGAACCAGCAGCGGAGCCAGCGGATCCAGACCGGGGACTTATCAGCCAACCTGTTAC
9042	CCAGCTTTTGTTCCCTTTAGTGAGCTTGTAAAGTTCATCCATACCGAG
9043	CCAGCTTTTGTTCCCTTTAGTGAGCTTATAAAGTTCGTCCATGCCAAG
9060	GGTTCTGGCGCTAGCATGGATTCAACAGAAGCAATTATTAAAG
9061	GTGAGTCGTGGGTTACTTGTACAAACTTCCGCCAGAG
9062	CCCACGACTCACTATAGGGCGA
9087	CCAGCTTTTGTTCCCTTTAGTGAGGGGTTACTTGTACAAACTTCCGCCAGAG

SEQUENCE *ptsG*^{early}*ΩTnFLXmsfgfp spec* (DB1567)

The *ptsG* gene is highlighted in cyan, *TnFLX* insertion sequences are highlighted in magenta, msfgfp is highlighted green and the spectinomycin resistance cassette (ant, aminoglycoside nucleotidyl transferase) is highlighted yellow.

5’
CAAATTGCCAAAGAAGTCATAGATATGATCAATGAAAAAGCGGGACTCTGCCTTCCGGAAGGGGAGATTGGTTTTATCGCTCTG
CATATCCATTCAGCGTTGACAAACCGTCCGCTGTCCGAGGTCAATCAGCACTCTCAGCTCATGGCGCAGCTTGTCGAGGTGATT
GAAGACTCATTTCAAATGAAAGTAAATAAGGAAAGTGTCAACTATTTGCGGCTGATCAGGCATATCAGATTTACAATAGAAAGAA
TTAAAAAAGAAGAACCGACTAAAGAACCAGAAAAATTAATGTTGTTATTGAAAAATGAATATCCGCTATGCTACAATACAGCTTGG
AAATTGATTAAAATCTTGCAGCAAACATTGAAGAAACCAGTTCATGAGGCGGAAGCGGTTTATCTGACGCTTCATCTGTACCGAT
TAACCAATAAAATTTCATAAATTCAGTTTATCCTTATAACGTGTTACTGATTCGATCAGGCATGAGTGATTGAGGGAAAAAAACGG
GAAGTTCATTCTCGTTCTTTTGCGCACCCAATTTTGCTCATGCCTTTTTGTTGTGTAAAAGGGCAAATGTAAACGGTTAAACTGGA
AGACTTACGCCTGTGAATTCGTTGTCATGATTTTTAGCTGTAAGGTCAGACTAGTAAAAAGAGGAGGTCAATTCTTATGTTTAAA
GCATTATTCGGCGTTCTTCAAAAAATTGGGCGTGCGCTTATGCTTCCAGTTGCGATCCTTCCGGCTGCGGGTATTTTGCTTGCG
ATCGGGAATGCGATGCAAAATAAGGACATGATTCAGGTCCTGCATTTCTTGAGCAATGACAATGTTCAGCTTGTAGCAGGTGTG
ATGGAAAGTGCTGGGCAGATTGTTTTCGATAACCTTCCGCTTCTTTTCGCAGTAGGTGTAGCCATCGGGCTTGCCAATGGTGAT
GGAGTTGCAGGGATTGCAGCAATTATCGGTTATCTTGTAACAGGTTGGCTGATAAGTCCCCGGTCTATGGTCTCCAAGGGAGAA
GAGTTATTTACAGGTGTAGTCCCGATCCTTGTAGAGCTTGATGGGGATGTCAACGGGCACAAATTTAGCGTCCGCGGGGAGGG
TGAGGGCGATGCTACAAACGGAAAATTGACGTTAAAATTCATTTGCACTACCGGCAAGCTTCCAGTTCCATGGCCAACGCTCGT
GACGACCCTCACCTACGGGGTGCAATGTTTTTCTCGTTATCCTGATCATATGAAACAACATGACTTTTTCAAATCAGCTATGCCT
GAAGGCTACGTGCAGGAGAGAACAATCTCCTTTAAGGACGACGGGACGTACAAAACACGCGCTGAGGTTAAATTTGAGGGGGA
CACTTTGGTGAATAGAATTGAGCTCAAAGGTATAGACTTTAAAGAGGATGGCAATATACTGGGTCACAAGCTCGAATATAACTTT
AACAGCCATAATGTATATATTACCGCAGATAAGCAGAAAAATGGGATAAAGGCGAACTTTAAAATACGCCATAACGTGGAAGATG
GCTCCGTACAACTTGCTGACCATTATCAACAGAATACACCAATTGGGGACGGCCCAGTGTTATTACCTGACAATCATTATCTTTC
CACACAATCAAAACTCTCTAAAGATCCTAATGAGAAACGTGATCATATGGTGCTGCTTGAGTTTGTGACTGCCGCTGGTATCACG
CACGGGATGGACGAGTTATATAAATAACCCACGACTCACTATAGGGCGAATTGGAGCTCCACCGCGGTGGCGGCCGCTCTAGA
ACTAGTGGATCCCCCGGGCTGCAGCTGGCGAATGGCGATTTTCGTTCGTGAATACATGTTATAATAACTATAACTAATAACGTAA
CGTGACTGGCAAGAGATATTTTTAAAACAATGAATAGGTTTACACTTACTTTAGTTTTATGGAAATGAAAGATCATATCATATATAA
TCTAGAATAAAATTAACTAAAATAATTATTATCTAGATAAAAAATTTAGAAGCCAATGAAATCTATAAATAAACTAAATTAAGTTTAT
TTAATTAACAACTATGGATATAAAATAGGTACTAATCAAAATAGTGAGGAGGATATATTTGAATACATACGAACAAATTAATAAAGT
GAAAAAAATACTTCGGAAACATTTAAAAAATAACCTTATTGGTACTTACATGTTTGGATCAGGAGTTGAGAGTGGACTAAAACCAA
ATAGTGATCTTGACTTTTTAGTCGTCGTATCTGAACCATTGACAGATCAAAGTAAAGAAATACTTATACAAAAAATTAGACCTATTT
CAAAAAAAATAGGAGATAAAAGCAACTTACGATATATTGAATTAACAATTATTATTCAGCAAGAAATGGTACCGTGGAATCATCCT
CCCAAACAAGAATTTATTTATGGAGAATGGTTACAAGAGCTTTATGAACAAGGATACATTCCTCAGAAGGAATTAAATTCAGATTT
AACCATAATGCTTTACCAAGCAAAACGAAAAAATAAAAGAATATACGGAAATTATGACTTAGAGGAATTACTACCTGATATTCCAT
TTTCTGATGTGAGAAGAGCCATTATGGATTCGTCAGAGGAATTAATAGATAATTATCAGGATGATGAAACCAACTCTATATTAACT
TTATGCCGTATGATTTTAACTATGGACACGGGTAAAATCATACCAAAAGATATTGCGGGAAATGCAGTGGCTGAATCTTCTCCAT
TAGAACATAGGGAGAGAATTTTGTTAGCAGTTCGTAGTTATCTTGGAGAGAATATTGAATGGACTAATGAAAATGTAAATTTAACT
ATAAACTATTTAAATAACAGATTAAAAAAATTATAAAAAAATTGAAAAAATGGTGGAAACACTTTTTTCAATTTTTTTGTTTTATTATT
TAATATTTGGGAAATATTCATTCTAATTGGTAATCAGATTTTAGAAAACAATAAACCCTTGCATAGGGGGATCTCGAGGGGGGGC
CCGGTACCAGCTTTTGTTCCCTTTAGTGAGGGGAGACCGGGGACTTATCAGCCAACCTGTTAATGAATGTATCCATGAGTGCGG
TTCTTCTTGCAAACGGAACCATTCCTTCGGATTCAGTTGAAAGAGCCAAGTTCTTTACGGAAAACCATCCTGCATATGTAAACAT
GCTTGGTATACCTACCTTGGCGACAGGGGTGTTCGGCGGTATTATCGTCGGTGTGTTAGCTGCATTATTGTTTAACAGATTTTAC
ACAATTGAACTGCCGCAATACCTTGGTTTCTTTGCGGGTAAACGTTTCGTTCCAATTGTTACGTCAATTTCTGCACTGATTCTGG
GTCTTATTATGTTAGTGATCTGGCCTCCAATCCAGCATGGATTGAATGCCTTTTCAACAGGATTAGTGGAAGCGAATCCAACCCT
TGCTGCATTTATCTTCGGGGTGATTGAACGTTCGCTTATCCCATTCGGATTGCACCATATTTTCTATTCACCGTTCTGGTATGAAT
TCTTCAGCTATAAGAGTGCAGCAGGAGAAATCATCCGCGGGGATCAGCGTATCTTTATGGCGCAGATTAAAGACGGCGTACAG
TTAACGGCAGGTACGTTCATGACAGGTAAATATCCATTTATGATGTTCGGTCTGCCTGCTGCGGCGCTTGCCATTTATCATGAAG
CAAAACCGCAAAACAAAAAACTCGTTGCAGGTATTATGGGTTCAGCGGCCTTGACATCTTTCTTAACGGGGATCACAGAGCCAT
TGGAATTTTCTTTCTTATTCGTTGCTCCAGTCCTGTTTGCGATTCACTGTTTGTTTGCGGGACTTTCATTCATGGTCATGCAGCTG
TTGAATGTTAAGATTGGTATGACATTCTCCGGCGGTTTAATTGACTACTTCCTATTCGGTATTTTACCAAACCGGACGGCATGGT
GGCTTGTCATCCCTGTCGGCTTAGGGTTAGCGGTCATTTACTACTTTGGATTCCGATTTGCCATCCGCAAATTTAATCTGAAAAC
ACCTGGACGCGAGGATGCTGCGGAAGAAACAGCAGCACCTGGGAAAACAGGTGAAGCAGGAGATCTTCCTTATGAGATTCTGC
AGGCAATGGGTGACCAGGAAAACATCAAACACCTTGATGCTTGTATCACTCGTCTGCGTGTGACTGTAAACGATCAGAAAAAGG
TTGATAAAGACCGTCTGAAACAGCTTGGCGCTTCCGGAGTGCTGGAAGTCGGCAACAACATTCAGGCTATTTTCGGACCGCGT
3'

SUPPLEMENTAL FIGURE LEGENDS

Figure S1: Parameters and gating for fluorescent events with the FACSAria II cell sorter. WT DK1042 cells were sorted using the FACSAria II and used to set the negative boundary for non-fluorescence (lower left panel). Each dot indicates an individual cell. Gray dots indicate a cell with fluorescence below gating threshold, green dots indicate a cell with fluorescence above a gating threshold. Next, DK1042 cells carrying the *pTnFLXmsfgfp* transposon delivery system were mutagenized, pooled and sorted using a 488 nm laser at 100 mW for collection. Results early in the sort are shown (lower middle panel) to indicate the rarity of green fluorescent cells, and late in the sort (lower right panel) to indicate the total number of green fluorescent cells acquired over the course of the separation. Cells with fluorescence below the threshold value were excluded as there were too many data points to show. The parameters used for the sorting shown inset above and were FSC-PMT at 300V, SCC at 269V, and B530/30 at 650V. All 3 parameters were set to a log threshold on SSC. Bright events were collected in LB broth and grown-up rolling overnight at 37°C.

Figure S2: CcpAΩmsfGFP fluorescence concentration colocalizes with DAPI staining. Micrograph of strain DB1701 expressing the CcpAΩmsfGFP fusion (false colored green, exposure 500 ms) and stained for nucleoids with DAPI (false colored blue, exposure 500 ms). The overlay of both indicates a nucleoid localization pattern for the CcpAΩmsfGFP fusion. Cells were imaged on 1% agarose pads and scale bar is 5 μm.

Figure S3: Membrane reporter localization is not negatively affected using different genetic backgrounds or mounting conditions. A) Fluorescence micrographs of the PtsGΩmsfGFP expressing expressed in ancestral 3610 (DB1567) and laboratory PY79 (DB2645) strains. Cells were imaged on 1% agarose pads, and the scale bar is 5 μm. B) Fluorescence micrographs of the PtsG-GFP expressing strain (DB1567) when mounted on either 1% agarose pads or treated with poly-L lysine adhesive. Scale bar is 5 μm. All msfGFP images were imaged with a 500 ms exposure time.

Figure S4: Timelapse imaging of FtsZ and the PtsG membrane reporter during microfluidic growth. Kymograph from 21 frames of imaging shown as a stack. The merged kymograph shows the mCherry-FtsZ (false colored red) and PtsGΩmsfGFP (false colored green). The separated channels show the equivalent plot profile of each signal intensity in sequential order. A merged image of the timeseries is shown on the right. The imaging was collected at 200 ms for mCherry and 300 ms for msfGFP.

Movie S1: 2 min interval imaging of membrane reporter and mCherry-FtsZ reporter. Strain, DB2118, with membrane reporter (false colored green, exposure 300 ms) and FtsZ reporter (false colored red, exposure 200 ms) imaged at 2 minute intervals in linear array channels. Scale bar is 5 μm.

Movie S2: Close up of 2 min interval imaging of membrane reporter and mCherry-FtsZ reporter.
Strain, DB2118, with membrane reporter (false colored green, exposure 300 ms) and FtsZ reporter (false colored red, exposure 200 ms) imaged at 2 minute intervals in linear array channel but a close up to visualize the trend of the septal formation and FtsZ recycling. Scale bar is 5 μm.

Figure S1

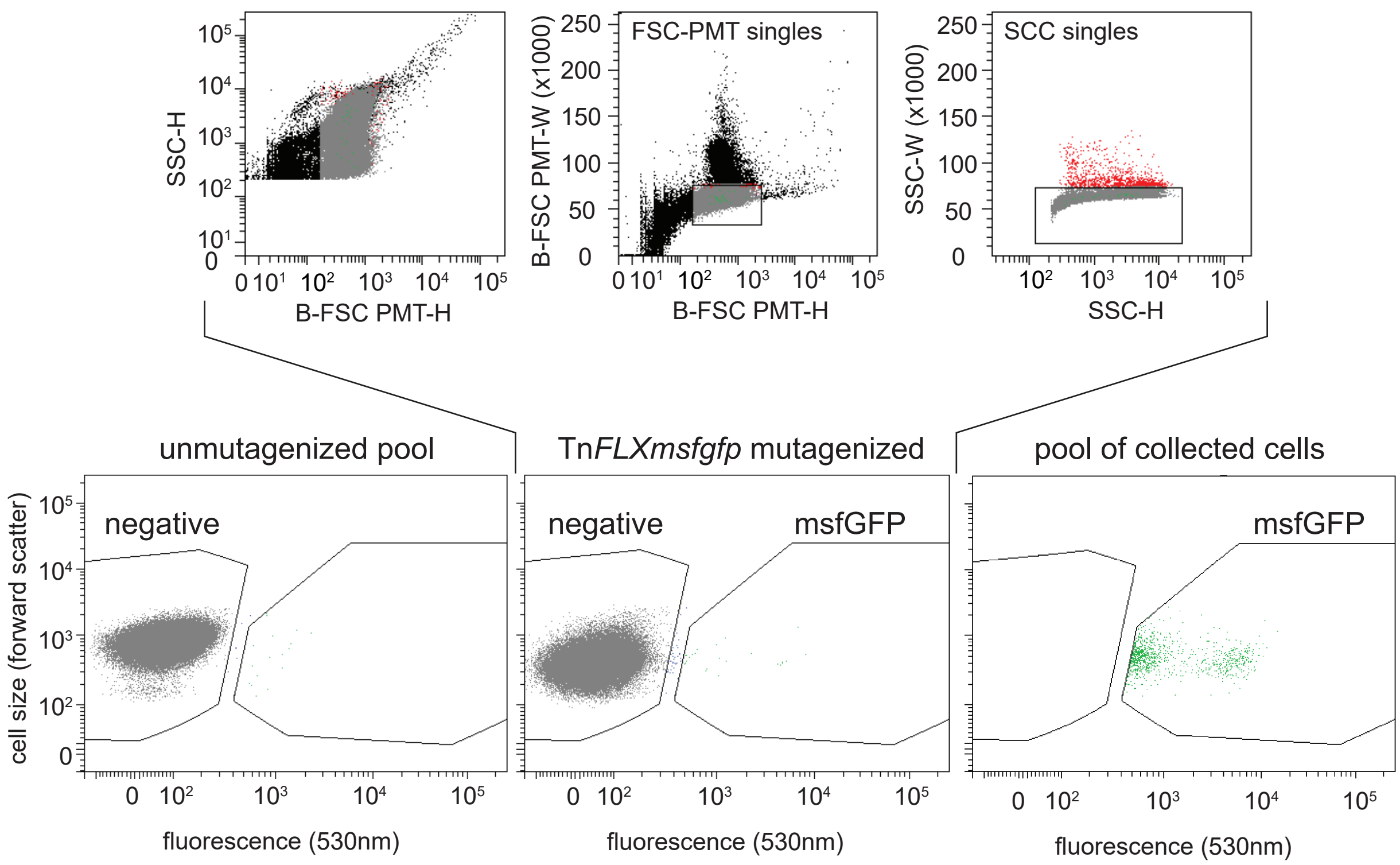

SSC-H
B-FSC PMT-H
FSC-PMT singles
B-FSC PMT-W (x1000)
B-FSC PMT-H
SCC singles
SSC-W (x1000)
SSC-H
unmutagenized pool
TnFLXmsfgfp mutagenized
pool of collected cells
negative
negative
msfGFP
msfGFP
cell size (forward scatter)
fluorescence (530nm)
fluorescence (530nm)
fluorescence (530nm)

Figure S2

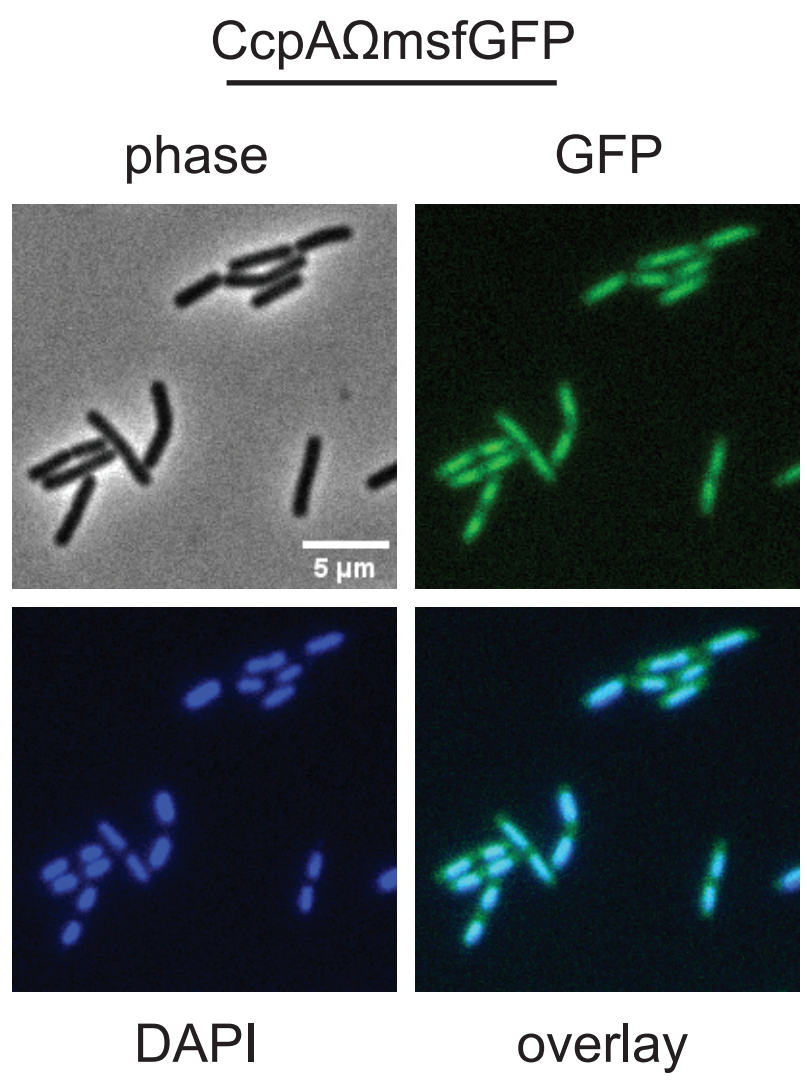

Figure S3

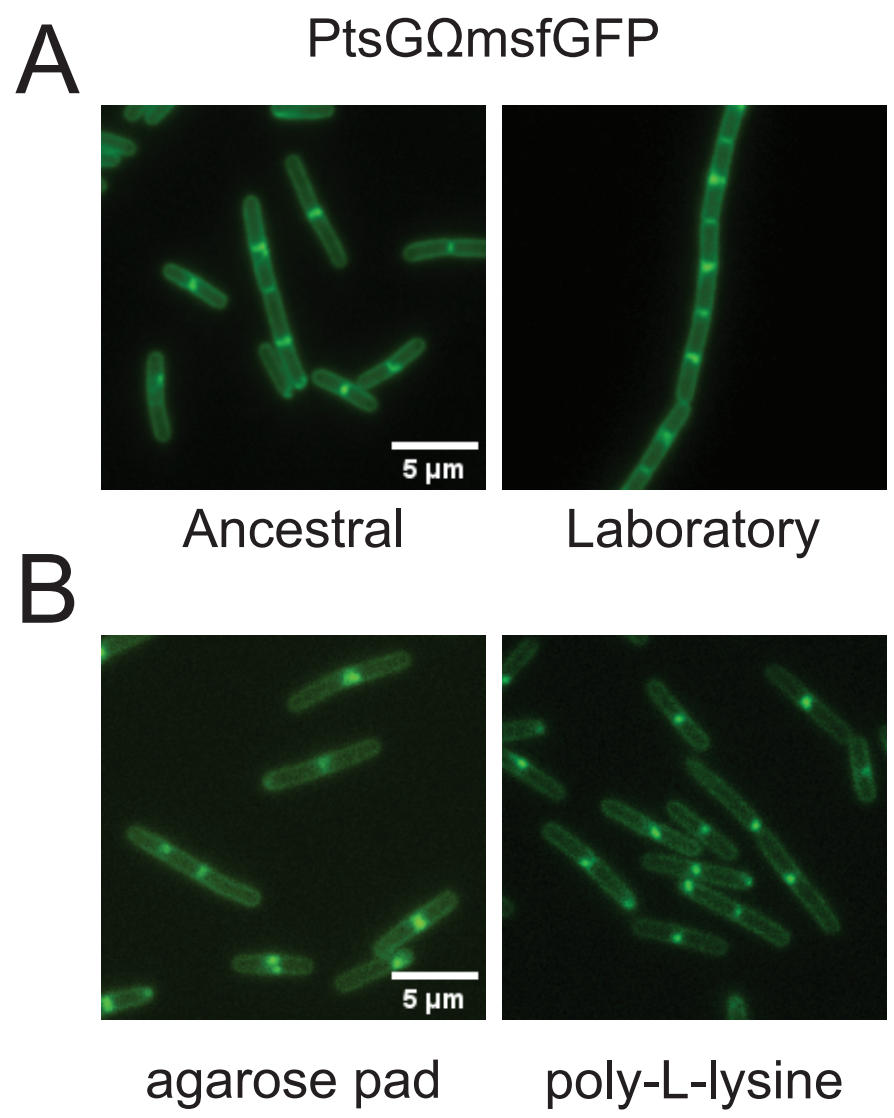
A
PtsGΩmsfGFP
5 µm
Ancestral
Laboratory
B
5 µm
agarose pad
poly-L-lysine

Figure S4

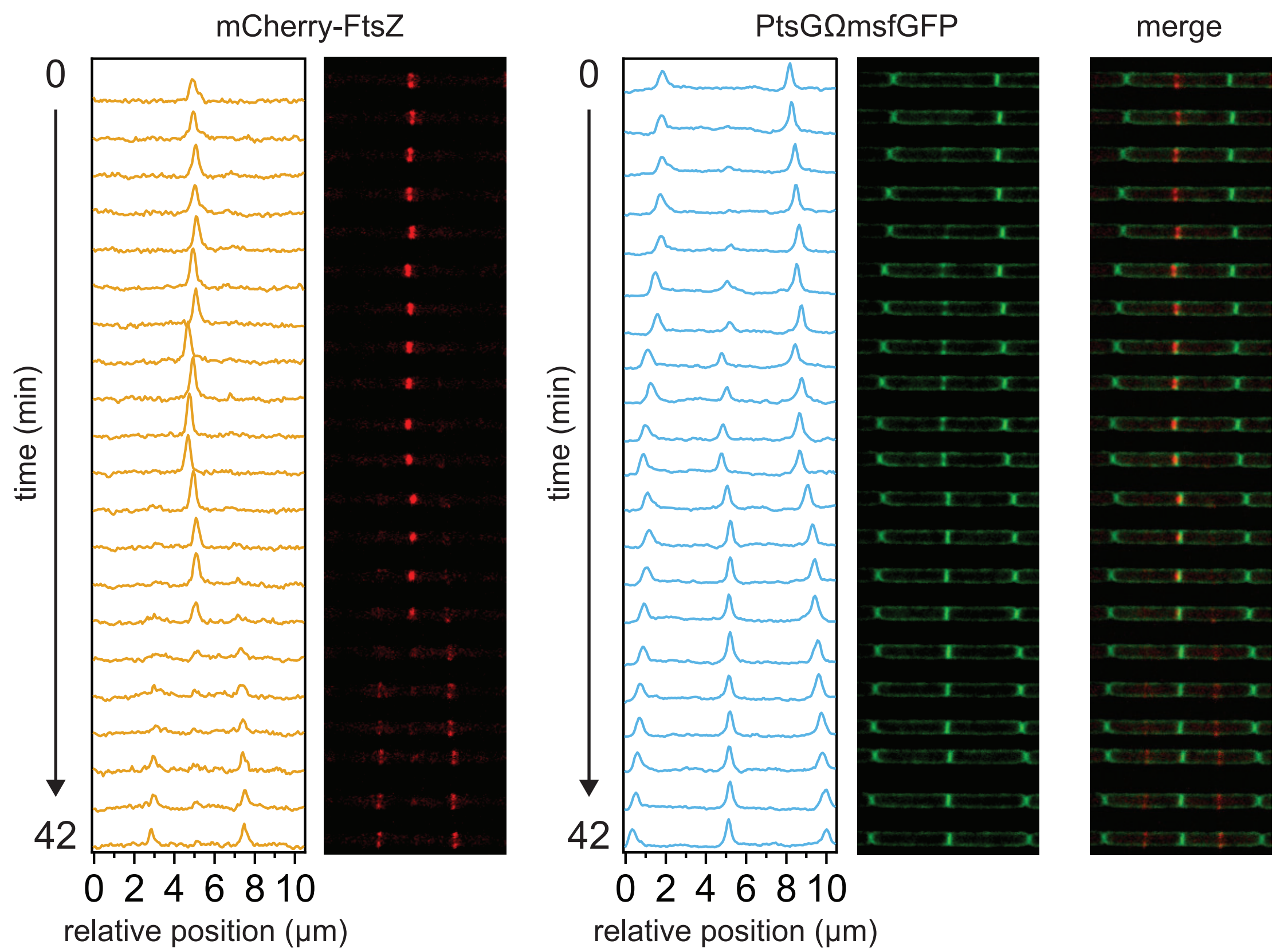
mCherry-FtsZ
PtsGΩmsfGFP
merge
0
42
time (min)
0
42
time (min)
0 2 4 6 8 10
relative position (µm)
0 2 4 6 8 10
relative position (µm)
